# Marburg and Sudan viruses elicit divergent interferon responses and cytokine storm signaling in Egyptian rousette bat macrophages

**DOI:** 10.3389/fimmu.2025.1686343

**Published:** 2025-10-17

**Authors:** Ivet A. Yordanova, Catherine E. Arnold, Nicolas Corrales, Jonathan C. Guito, Angelika Lander, Lay Teng Ang, Jonathan S. Towner, Joseph B. Prescott

**Affiliations:** ^1^ Centre for Biological Threats and Special Pathogens, Robert Koch Institute, Berlin, Germany; ^2^ Diagnostic Systems Division, US Army Medical Research Institute of Infectious Diseases, Ft. Detrick, MD, United States; ^3^ Viral Special Pathogens Branch, Centers for Disease Control and Prevention, Atlanta, GA, United States; ^4^ Stanford Institute for Stem Cell Biology and Regenerative Medicine, Stanford University, Stanford, CA, United States; ^5^ Department of Urology, Stanford University, Stanford, CA, United States

**Keywords:** Marburg virus, Sudan virus, filovirus, Egyptian rousette bat, reservoir, macrophage, innate immune response

## Abstract

**Introduction:**

Egyptian rousette bats (ERBs) are the only known natural reservoir of Marburg virus (MARV), etiologic agent of a highly-pathogenic zoonotic viral hemorrhagic fever. Evolutionary adaptations in ERBs allow for fine-tuned discrete pro-inflammatory immune responses that control MARV infection, yet permit population-level viral maintenance.

**Methods:**

To look for exclusive co-adapted responses between ERBs and MARV, we compared macrophage (MΦ) responses to MARV and Sudan virus (SUDV), a related filovirus not hosted by ERBs. We queried whether MARV counters normal ERB MΦ responses, illuminating co-adapted host responses not observed upon infection with SUDV, which fails to establish a productive infection and is efficiently immunologically cleared by ERBs.

**Results:**

We observed stark differences in MΦ transcriptional responses to MARV and SUDV, including differences in type I and III interferon (IFN)-related genes, cytokines, chemokines, cell growth and proliferation genes. We show for the first time that while MARV-infected bat MΦs undergo muted IFN responses and cytokine storm signaling, SUDV induces unperturbed type I and III IFN gene expression, stronger cytokine and chemokine responses resembling typical host responses to a foreign viral pathogen.

**Discussion:**

Our findings corroborate growing evidence of unique coevolutionary relationships between bats and the specific viruses they harbor.

## 1 Introduction

Bats possess an exceptional kaleidoscope of evolutionary adaptations, including powered flight, unique among mammals, species diversity second only to rodents, and diverse diets including but not limited to blood, nectar, fruit, insects, and fish. Bats are increasingly being recognized as important reservoirs of high-consequence viral zoonoses, including various henipaviruses, rhabdoviruses and filoviruses (1–3). Due to their ability to transmit zoonotic pathogens to humans, domestic animals or other wildlife, several bat species represent a significant spillover risk. Hence, understanding how these zoonotic viruses have co-adapted to their natural reservoirs and are ecologically maintained is of paramount importance.

The ability of some bats to host zoonotic viruses otherwise pathogenic in humans and non-human primates (NHPs) has fostered rising interest in bat biology, ecology, viral diversity, and immune system evolution (4–10). The findings from many of these studies illustrate that different bats have evolved diverse and highly-specific molecular mechanisms that likely contribute to their ability to tolerate and transmit viral pathogens by striking a fine-tuned balance of inducing sufficient antiviral immune responses to clear infection without aberrant tissue-damaging inflammatory processes.

Among the best characterized bat-zoonotic pathogen relationships to date is that of Egyptian rousette bats (ERBs, *Rousettus aegyptiacus*), the only verified natural reservoir of the orthomarburgviruses Marburg (MARV) and Ravn (RAVV) (11–16). Unlike orthomarburgviruses, the natural reservoir of pathogenic orthoebolaviruses like Ebola virus (EBOV; species *Orthoebolavirus zairense*) and Sudan virus (SUDV; species *Orthoebolavirus sudanense*) remains unknown, even though bat species other than ERBs are suspected as natural reservoirs (17–20). Along with EBOV and SUDV, MARV is an etiologic agent of sporadic outbreaks of viral hemorrhagic fever across Sub-Saharan Africa, with case fatality rates ranging from 40% to 90% (21, 22). Humans and NHPs infected with EBOV, SUDV or MARV typically develop initial non-specific flu-like symptoms, including high fever, muscle and joint pain, often followed by the rapid development of severe neurologic and hemorrhagic symptoms (23–25). In contrast, ERBs support MARV replication in diverse tissues and shed infectious virus in the absence of signs of inflammatory disease, highlighting their reliance on a refined and highly specific co-adapted relationship with MARV (15, 26, 27).

Previously, we successfully differentiated bone marrow-derived dendritic cells (bmDC) from ERBs, demonstrating that these cells support low-level MARV infection and intracellular replication. MARV-infected DCs elicited a balanced response involving upregulated canonical antiviral signaling genes and suppressed proinflammatory cytokine/chemokine gene expression (28). Similarly, CD14^+^ monocyte-like cells isolated from the spleens of MARV-infected ERBs support viral replication and display transient upregulation of genes associated with type I IFN responses, viral restriction, and anti-inflammatory signaling pathways (29). In the liver, MARV-infected ERBs harbor discrete foci of inflammation in the absence of notable tissue pathology elsewhere (27). In infected bats, viral RNA is clearly detectable in Iba1^+^ mononuclear phagocytes in the liver, as well as in follicular DC-like cells in axillary lymph nodes, underlining the specific cell tropism of MARV for host MΦs and DCs (27). Similar host cell tropism is also observed in humans and NHPs, where filoviruses induce significant tissue-damaging proinflammatory cytokine and chemokine release in MΦs, while DCs undergo arrested maturation and display dysregulated antigen presentation functions (30–36).

Contrasting their reservoir competence for MARV, ERBs are generally refractory to orthoebolaviruses like EBOV, Bundibugyo virus (BDBV), Taï Forest virus (TAFV) or Reston virus (RESTV) (26). Only low levels of SUDV viral RNA have been detected in select tissues of experimentally infected ERBs, in the absence of viral shedding. Comparative analysis of MARV and SUDV viral loads in liver, spleen and kidney tissues shows ERBs control SUDV replication faster than MARV, indicating differential control of the two filoviruses (26). However, the underlying innate immune mechanisms potentially contributing to this divergent control of filovirus infections in these bats remain unknown. Directly comparing ERB MΦ responses to MARV and SUDV infections therefore offers an invaluable opportunity to elucidate specific host responses that have evolved during co-adaptation that allow for the development of a productive MARV infection, but a non-productive “dead-end” infection with SUDV. By leveraging our ability to generate bone marrow-derived MΦs, we were able to show that MARV evades specific features of ERB immunity, including macrophage activation and type III IFN responses, both of which are induced by SUDV, likely contributing to the ability of these bats to efficiently combat SUDV. In contrast, MARV is able to evade immunity to prolong replication and infection by relying on a complex combination of viral protein antagonism patterns and host cell cytoskeletal changes unique to ERBs.

## 2 Results

### 2.1 ERB bone marrow cells differentiate into MΦs in response to recombinant bat M-CSF

**Figure 1:**
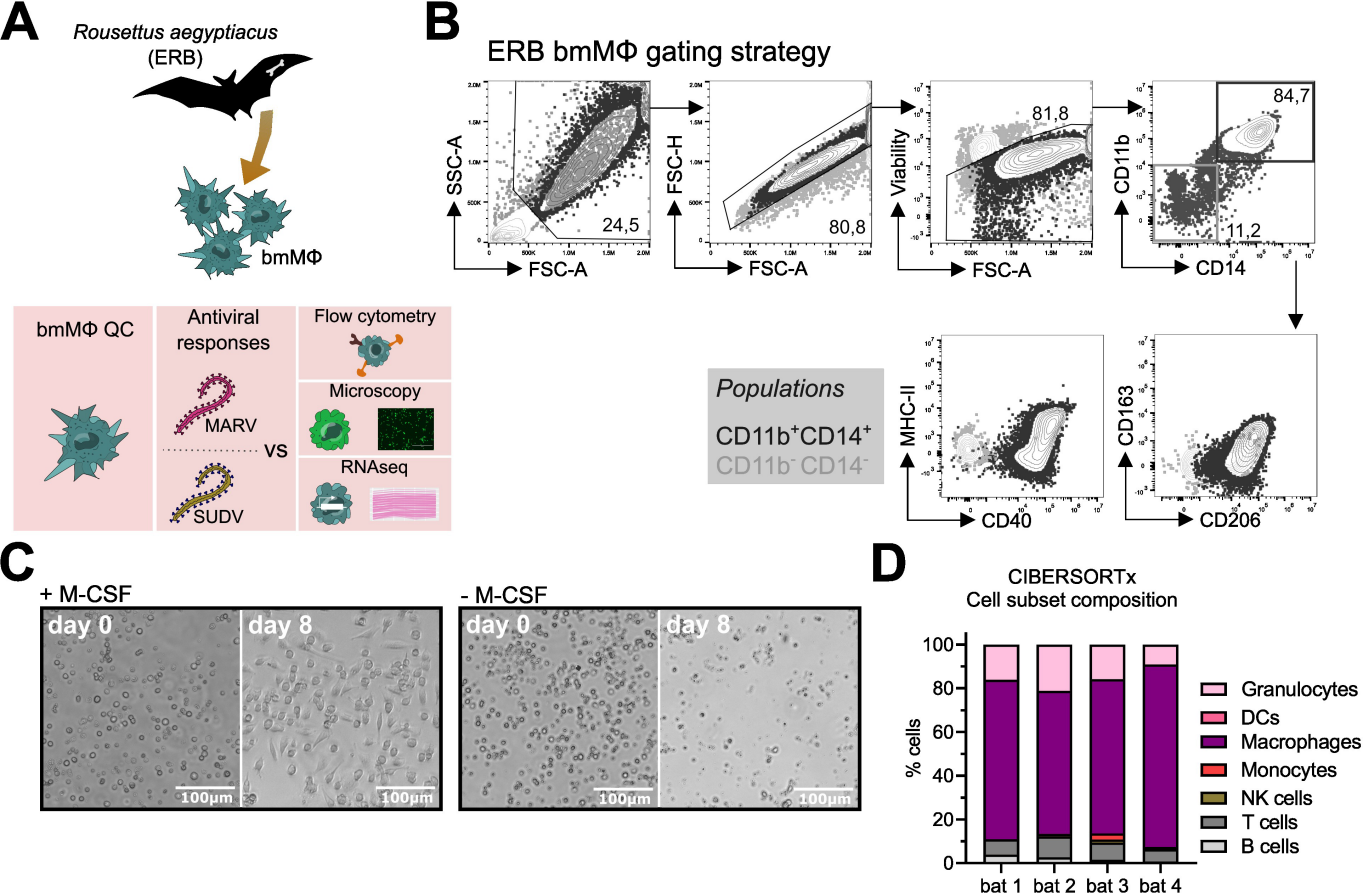
Differentiation and initial characterization of Egyptian rousette bat (ERB) bone marrow-derived macrophages (bmMΦs). **(A)** Graphical summary of the experimental framework. **(B)** Example brightfield microscopy images illustrating the morphology of bone marrow cell cultures following 8 days of *in vitro* differentiation with (left) or without (right) recombinant ERB macrophage colony-stimulating factor (M-CSF). **(C)** Gating strategy and example contour plots showing the identification of CD11b^+^CD14^+^ bmMΦs (black gate) via flow cytometry and their expression of surface markers CD40, MHC-II, CD163 and CD206, overlaid with the expression of the same markers in undifferentiated CD11b^-^CD14^-^ cells (grey gate). **(D)** Percentages of CD11b^+^CD14^+^ and CD11b^-^CD14^-^ cells among live singlets in M-CSF-differentiated bone marrow cultures. **(E)** Median fluorescence intensities (MFI) of surface markers CD163, CD206, MHC-II and CD40 expressed on CD11b^+^CD14^+^ bmMΦ and CD11b^-^CD14^-^ non-bmMΦ cells after 8 days of culture. **(F)** Representative confocal microscopy images of mock, MARV-ZsG and SUDV-ZsG-infected bmMΦs. Scale bars correspond to 16µm. Cell nuclei are stained in blue (DAPI) and actin filaments in magenta (Phalloidin). Virus-infected cells contain fluorescent ZsGreen signal. The smaller panels on the right illustrate zoomed in images of the cells marked with dotted squares in the main panels on the left. **(G)** CIBERSORTx analysis of the baseline cell subset composition of ERB-derived bmMΦs. The analysis used the transcriptional profile of mock-infected bmMΦs compared against known signatures identified for human marker genes using a software-defined signature matrix to calculate the proportions of cell types. The results in **(D)** and **(E)** are pooled from two independent experiments with four biological replicates. Statistical analysis was performed using a Wilcoxon Signed Rank test **(D)** or a Šídák’s multiple comparison test **(E)**. ***p < 0.001.

Dysregulation of host MΦs is a classical feature of filovirus disease. We therefore sought to examine the immune response profile of filovirus-infected ERB MΦs. For this, we optimized our existing approach for ERB bmDC differentiation into a novel protocol to reproducibly generate bmMΦs *in vitro* (**Figure 1A**). Bone marrow cells isolated from naïve bats were cultured in medium containing ERB-specific recombinant macrophage colony-stimulating factor (M-CSF; Kingfisher Biotech). In the presence of M-CSF, bone marrow cells consistently developed heterogeneous morphology after 8 days, but maintained consistent adherence properties and displayed dendrites, typical morphological features of macrophages (**Figure 1B**, **Supplementary Figure S1A**). In contrast, cultures without M-CSF contained only small, non-adherent cell-like particles and debris (**Figure 1B**).

To confirm the phenotype of our ERB bmMΦs, we quantified the cell surface expression of canonical MΦ markers, including myeloid cell markers CD11b and CD14, the scavenger receptor CD163, the mannose receptor CD206, the antigen presentation receptor MHC-II and the costimulatory marker CD40 (**Figures 1C-E**). Bone marrow cells cultured with M-CSF generated variable proportions of bmMΦs from individual bats, averaging 65-85% of myeloid CD11b^+^CD14^+^ cells (**Figure 1D** **,** **Supplementary Figures S1B–E**). Contrasting with CD11b^-^CD14^-^ cells (non-bmMΦ), the CD11b^+^CD14^+^ population (bmMΦ) had higher surface expression of CD163, CD206 and MHC-II, while CD40 expression in bmMΦs was almost 10^4^-fold higher than in non-bmMΦ (**Figure 1E**).

Host MΦs are among the first targets of filoviruses *in vivo*. To assess whether ERB-derived bmMΦs are susceptible to MARV and SUDV, and to better capture their morphology at baseline and following initial filovirus infection, bmMΦs were infected with recombinant MARV or SUDV viruses expressing a green fluorescent protein (ZsGreen, ZsG) or were left uninfected. Using high-resolution confocal microscopy and staining of cell nuclei (DAPI, blue) and the cytoskeleton (Phalloidin, magenta), we observed classic macrophage morphology (**Figure 1F**). In MARV-infected cells we observed granular foci of strong ZsG signal, possibly as a result of partially incomplete cleavage of the NP-ZsG fusion protein as previously reported (37). The cells were also readily susceptible to SUDV, evidenced by the presence of strong but more diffuse cytoplasmic ZsG signal in line with its VP40-ZsG fusion protein construct and typical assembly mechanism of SUDV in host cells (38, 39). Together, these microscopy findings illustrate that ERB-derived bmMΦs display a classical macrophage morphology and demonstrate evidence of characteristic viral replication and assembly.

Finally, we performed bulk RNA sequencing of freshly-differentiated bmMΦs to assess their baseline transcriptional profile as an additional quality control step. Using the complete transcriptional profile of the cells, we assessed their cell culture composition using CIBERSORTx and could show that our cultures were predominantly classed as “macrophages” based on their complete gene expression profile (**Figure 1G**).

### 2.2 MARV replication dynamics differ from SUDV in ERB innate immune cells

To detect differences in viral transcription efficiency between MARV and SUDV, ERB bmMΦs were infected with wild-type MARV or SUDV, or recombinant fluorescent ZsG viruses (MARV-ZsG or SUDV-ZsG) to monitor the kinetics of viral infection, replication and progeny production. Cells were infected with each virus at a multiplicity of infection (MOI) of 2 (measured on Vero E6 cells), and samples were collected over 3 days for RNA sequencing and qRT-PCR (wild-type viruses) or were observed microscopically (ZsG-expressing viruses).

**Figure 2:**
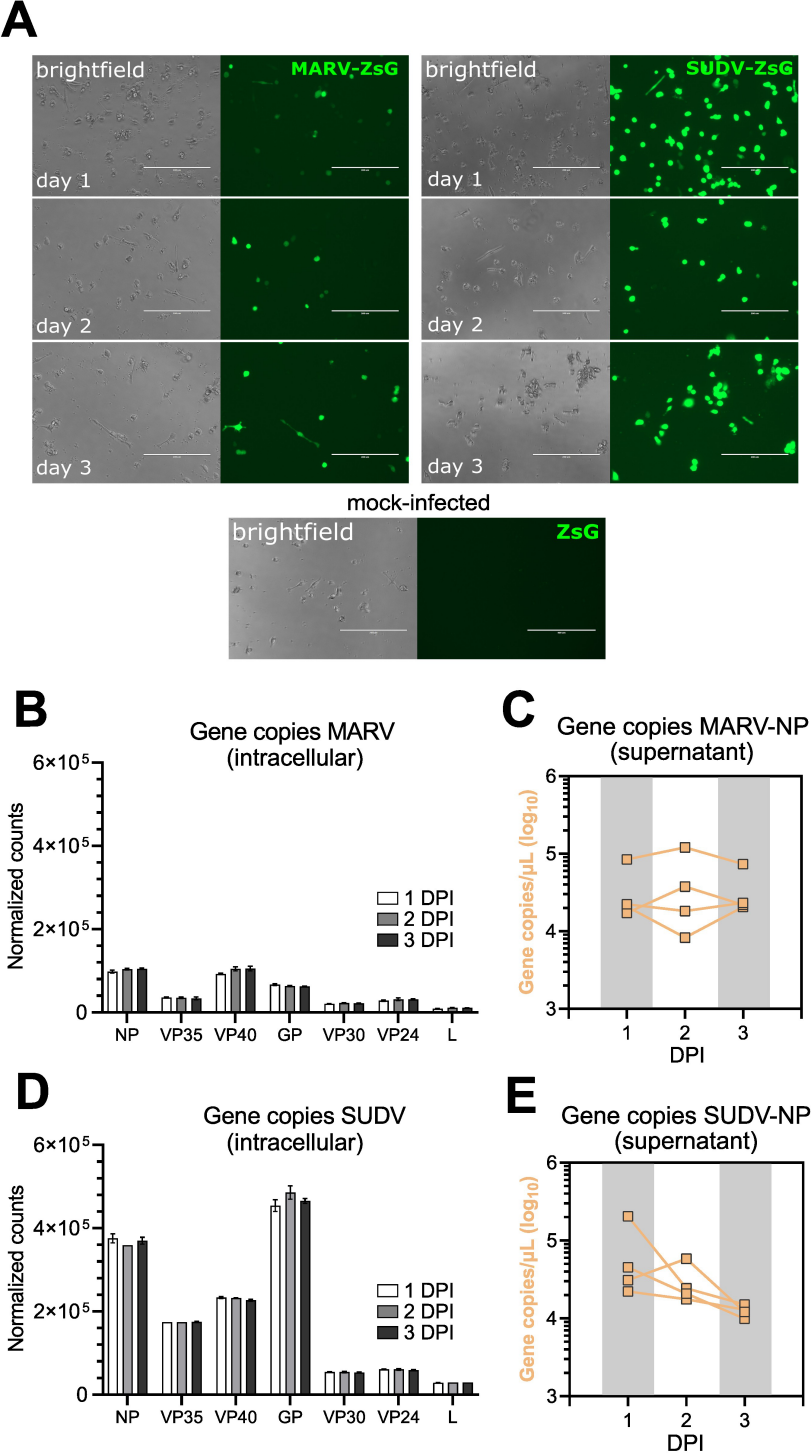
Intracellular virus replication and progeny production in ERB bmMΦs. **(A)** Fluorescent ZsGreen expression in MARV-ZsG-infected, SUDV-ZsG-infected and mock-infected ERB bmMΦs at 1, 2, and 3 DPI. Scale bars correspond to 200 µm. **(B)** Normalized counts of MARV genes at 1, 2 and 3 DPI in bmMΦs. **(C)** Gene copy counts of MARV-NP in cell culture supernatants. **(D)** Normalized counts of SUDV genes at 1, 2 and 3 DPI in bmMΦs. **(E)** Gene copy counts of SUDV-NP in cell culture supernatants. The data in **(B–E)** are pooled from two independent experiments with four biological replicates. Statistical analysis was performed using Tukey’s multiple comparison’s test. The bar plots in **(B, D)** illustrate mean ± SD.

Cells infected with either virus displayed clear signs of viral protein transcription over the course of the 3-day infection, evidenced by the presence of ZsG signal in virus-infected bmMΦ cultures (**Figure 2A**). Bulk RNAseq analysis revealed that SUDV-infected cells harbored higher intracellular viral gene copy numbers of NP (3.7-fold higher), VP35 (4.8-fold higher), VP40 (2.5-fold higher) and GP (7-fold higher) compared with MARV-infected cells, indicative of higher viral replication of SUDV in these cells and in line with the stronger ZsG signal observed (**Figures 2A, B, D**). Surveying viral progeny production in cell culture supernatants, cells from individual bats maintained overall stable numbers of MARV-NP gene copies/µL supernatant between 1–3 DPI. In contrast, SUDV-infected cells showed a trend for decreasing viral progeny production between 1 DPI and 3 DPI, suggestive of efficient control of infection compared with MARV (**Figures 2C, E**).

Considering the difference in virus replication between MARV and SUDV in ERB bmMΦs, next we sought to test whether intrinsic differences in replication between the two viruses explain this observation. For this, we infected ERB kidney-derived immortalized RoNi cells and Vero E6 cells with MARV-ZsG and SUDV-ZsG. At 1, 2 and 3 DPI each cell line was surveyed via flow cytometry and qRT-PCR to quantify the percentage ZsG-positive cells and viral RNA in cell culture supernatants (**Supplementary Figures S2A–D**). Overall, MARV-ZsG and SUDV-ZsG replicated similarly in Vero E6 cells. In contrast, we observed significantly fewer ZsG^+^ cells at 2–3 DPI in MARV-infected RoNi cells, diverging from the SUDV-ZsG replication in RoNi cultures, which was comparable to that observed in Vero E6 cells (**Supplementary Figures S2A–C**). Together, these findings highlight that instead of virus-intrinsic differences, the differential replication of MARV and SUDV is host-intrinsic, likely as a result of specific co-evolutionary adaptations between ERBs and MARV.

### 2.3 ERB bmMΦs mount transcriptionally distinct responses to general immune stimulation and filoviruses

**Figure 3:**
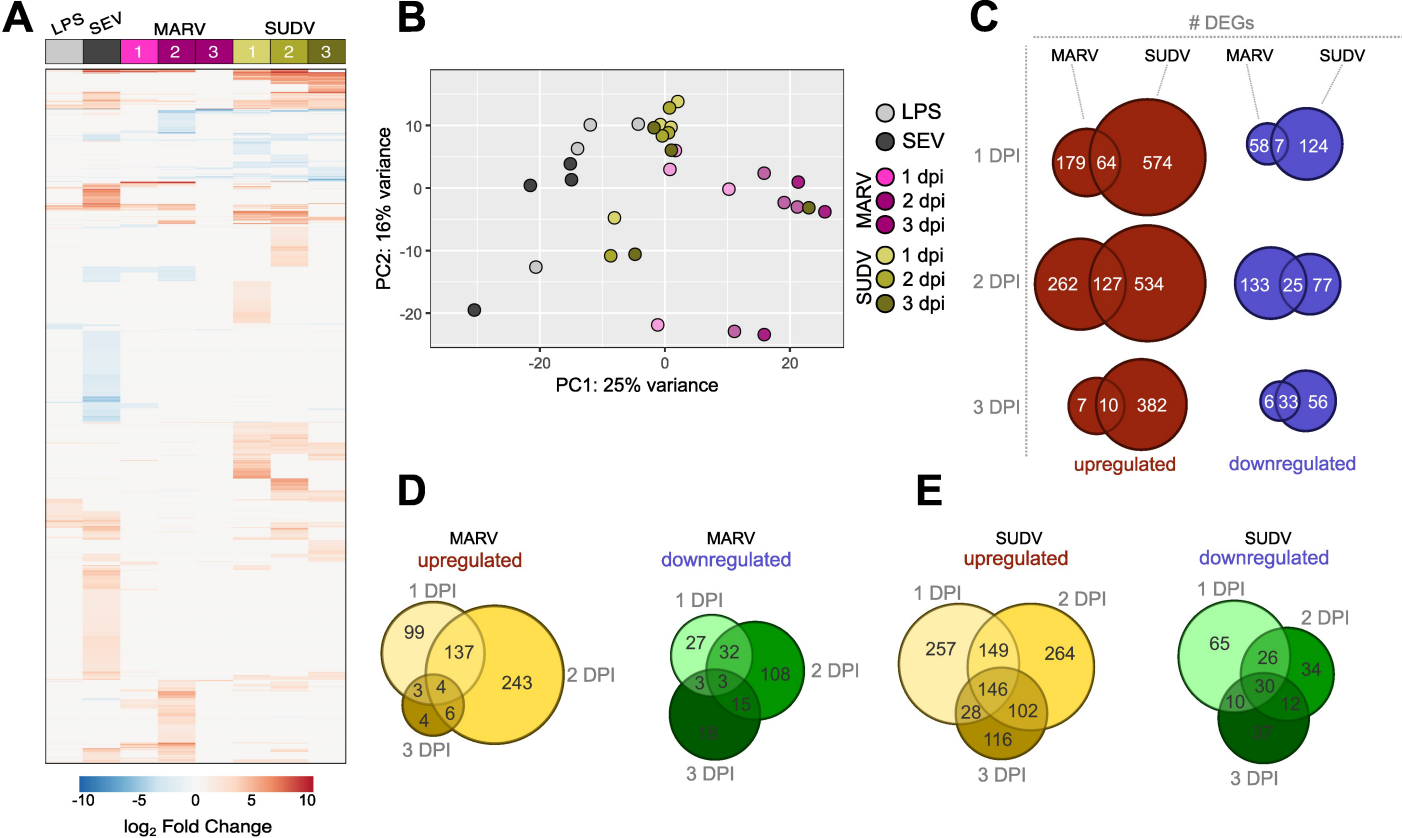
Global transcriptional responses of bat bmMΦs to stimulation and viral infection. **(A)** Global heatmap of differentially expressed genes (DEG) in ERB bmMΦs after 1 day of LPS restimulation and SeV infection, and at 1, 2 and 3 DPI with MARV or SUDV. DEGs were defined as genes with a *p-adj* < 0.05 and log_2_-fold change ≥ ± 1.5 against mock-infected negative controls. **(B)** Principal component analysis (PCA) plot of LPS-treated, SeV-infected and filovirus-infected bmMΦs, based on the expression levels of the top 500 most highly-expressed genes in each treatment group. **(C)** Venn diagram of the total numbers of significantly upregulated (red) and downregulated (blue) DEGs identified uniquely for MARV, SUDV or shared in both viral infections at 1, 2 and 3 DPI. **(D)** Venn diagram of the total number of upregulated (left) and downregulated (right) DEGs unique to each time point or shared between time points of infection in MARV-infected bmMΦs. **(E)** Venn diagram of the total number of upregulated (left) and downregulated (right) DEGs unique to each timepoint or shared between timepoints of infection in SUDV-infected bmMΦs.

Next, we applied bulk RNA sequencing to profile the transcriptional responses of bmMΦs to both general stimulation and filovirus infections, expanding the depth and breadth of our understanding of ERB innate immune responses at the cellular level. Similar to bmDCs (28), ERB-derived bmMΦs displayed a clear transcriptional response distinct between general immune agonists like bacterial lipopolysaccharide (LPS) and Sendai virus (SeV), and the two filoviruses (**Figure 3A**). MARV induced the differential expression of overall smaller clusters of genes than SUDV, mostly at 1 DPI and 2 DPI, while SUDV induced the consistent differential expression of larger gene sets throughout all three timepoints (**Figure 3A**). In response to LPS, bmMΦs upregulated various transcriptional factors (*STAT4*), proinflammatory cytokines (*TNF, IL1A, IL6, IL12B, IL23)*, cell migration receptors (*CCR7, ITGB8*) and chemokines (*CXCL6, CCL22*) (**Supplementary Figure S3A**). In contrast, SeV infection induced canonical antiviral IFN-associated genes like *IFNB1, ISG20* and *IFIT3*, several chemokine and chemokine receptor genes (*CCL5, CXCL11, CCR3, CCR7*), proinflammatory cytokines (*IL6*) and activation markers (*CD82, CD163, CD207*) (**Supplementary Figure S3B**).

Principal component analysis based on the top 500 most highly differentially expressed genes confirmed the treatment and infection-specific responses across the four individual bats (**Figure 3B**). ERB bmMΦs clustered predominantly by treatment (LPS) or virus infection (SeV/MARV/SUDV), with temporal differences in clustering mostly evident for MARV-infected samples, while most SUDV samples clustered together independent of day of infection, in line with the continuous differential expression of large gene sets shown in the DEG heatmap (**Figures 3A, B**). SUDV induced almost 5-fold more unique upregulated DEGs (574 *vs* 179) and twice the number of unique downregulated differentially expressed genes (DEGs) (124 *vs* 58) than MARV as early as 1 DPI. While the expansion of both unique and shared DEGs for MARV was only transient and significantly contracted by 3 DPI, SUDV-infected bmMΦs maintained stable differential expression of large sets of both upregulated and downregulated DEGs unique to SUDV throughout the 3-day infection (**Figure 3C**). Comparison of the numbers of unique and shared DEGs per timepoint of infection further revealed that both MARV and SUDV induced unique gene sets at 1, 2 or 3 dpi in addition to shared gene signatures at 1-2, 2–3 and 1–3 dpi (**Figures 3D, E**).

### 2.4 bmMΦs initiate disparate host cell transcriptional responses to MARV and SUDV

**Figure 4:**
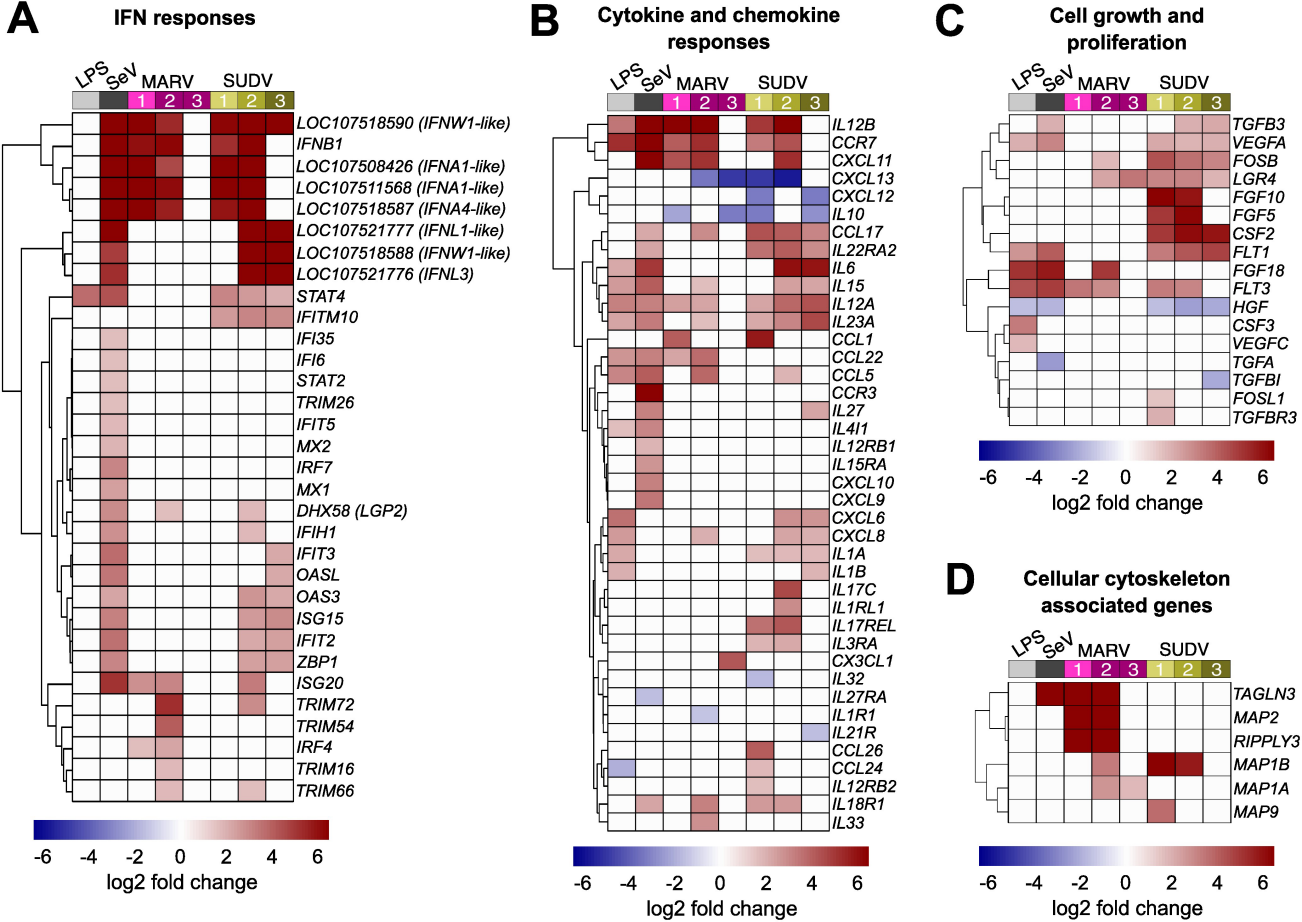
Immune-related gene expression profile of ERB-derived bmMΦs. Heatmaps of **(A)** IFN response DEGs; **(B)** Cytokine and chemokine DEGs; **(C)** Cell growth and proliferation-related DEGs and **(D)** Host cell cytoskeleton-associated DEGs in ERB bmMΦs after 1 day of LPS restimulation and SeV infection, and at 1, 2 and 3 DPI with MARV or SUDV. DEGs were defined as genes with a *p-adj* < 0.05 and a log_2_-fold change ≥ ± 1.5. Each treatment group or timepoint includes pooled data from four individual bats. Each heatmap is shown as log_2_-fold change against mock-infected negative controls.

Significant IFN-associated host cell transcriptional responses to both filoviruses consisted almost exclusively of upregulated genes, whose differential expression was mostly limited to 1–2 DPI for MARV, and 1–2 DPI or 2–3 DPI for SUDV. Within the first two days of infection with either filovirus, ERB bmMΦs upregulated an identical cluster of type I IFN genes, including *IFNB1*, two *IFNA*-like, an *IFNA4*-like and an *IFNW1*-like gene – a response mirrored in SeV-infected cells. Another *IFNW1*-like gene and two type III IFN genes (*IFNL1*-like and *IFNL3*) were upregulated only in response to SeV and SUDV at 2 and 3 DPI (**Figure 4A**). Beyond type I IFNs, MARV infection induced overall muted IFN-associated gene expression in bmMΦs, characterized mostly by transient upregulation of *ISG20* and *IRF4* at 1 and 2 DPI, and the upregulated expression of four TRIM protein-coding genes at 2 DPI (*TRIM16, TRIM54, TRIM66* and *TRIM72*). SUDV induced a stronger shift in gene expression, including the stable upregulation of *STAT4* and *IFITM10* between 1–3 DPI, a delayed upregulation of multiple ISGs (*IFIH1, IFIT2, IFIT3, OASL, OAS3, ISG15, ISG20, ZBP1)*, but only transient upregulation of *TRIM66* and *TRIM72* at 2 DPI (**Figure 4A**).

Alongside antiviral IFN responses, ERB bmMΦs displayed diverse differential gene expression of various cytokines and chemokines in response to both filoviruses. Between 1 and 2 DPI, both MARV and SUDV upregulated the expression of *IL12B, CCR7* and *CXCL11*, reflecting a similar response in LPS-stimulated and SeV-infected cells. In contrast, the anti-inflammatory cytokine *IL10*, and the chemokines *CXCL12* and *CXCL13* were strongly downregulated in response to both MARV and SUDV, but remained unchanged following LPS stimulation or SeV infection, indicative of filovirus-specific gene suppression (**Figure 4B**). MARV infection induced a muted sporadic upregulation at either 1 DPI or 2 DPI of several chemokines (*CCL1, CCL5, CCL17, CCL22, CXCL8, CXCL11*) and cytokines (*IL12A, IL15, IL23A, IL33*). In contrast, SUDV infection upregulated more sustained expression of *CCL17*, paralleled by a transient upregulation of more proinflammatory cytokines and chemokines at either 1–2 DPI (*CCL1, IL17C, CCL24, CCL26*) or 2–3 DPI (*IL6, IL15, IL12A, IL23A, CXCL6, CXCL8*) (**Figure 4B**).

In parallel with the observed expression profiles of immune-related genes, filovirus-infected bmMΦs shifted their expression of several cell growth and proliferation-associated genes. SUDV induced the sustained upregulation of genes like *FOSB, LGR4, FGF5, FGF10, CSF2, FLT1* and *FLT3*. In contrast, MARV-infected cells underwent weaker transient upregulation of *FOSB*, *LGR4*, *FGF18* and *FLT3*. Interestingly, the hepatocyte growth factor *HGF*, whose gene expression was significantly downregulated in LPS-treated, SeV-infected and SUDV-infected cells remained unchanged in response to MARV, indicating potential MARV-specific regulation of growth factor expression in bat innate immune cells, potentially to limit hepatic injury. (**Figure 4C**).

Viruses rely on the host cell machinery not only for viral genome replication, but also for trafficking to sites of replication, shuttling intermediate viral products between sites of assembly within the cytoplasm and egress of newly assembled viral particles. Considering the pronounced differences in MARV and SUDV replication in ERB bmMΦs, we next surveyed the top DEGs in filovirus-infected cells for genes associated with host cell actin filament organization and polymerase activity. Among these, we found four genes encoding microtubule associated proteins (*MAP*s), *RIPPLY3* encoding RNA polymerase II, and *TAGLN3* associated with actin filament organization and RNA polymerase II transcription. MARV infection upregulated the expression of five of these DEGs (*MAP1A, MAP1B, MAP2, RIPPLY3* and *TAGLN3*), while SUDV only induced the upregulation of *MAP1B* and a transient increase of *MAP9* at 1 DPI. Considering the sustained low-level replication of MARV, these findings could indicate virus-induced alterations of the ERB cell cytoskeleton that contribute to a slower but more sustained viral replication and progeny production that ultimately translates in the ability of wild ERBs to maintain low-level MARV infections long enough for virus transmission to other bats and long-term maintenance at the population level year-long (**Figure 4D**). In contrast, SUDV could be maladapted to utilize the ERB intracellular machinery for efficient virus progeny assembly, trafficking and egress.

### 2.5 ERB bmMΦs experience differential antagonism by filovirus-encoded proteins

Filoviruses have evolved various strategies to either evade or antagonize host innate immunity through distinct mechanisms driven mostly by filoviral VP24, VP35 and VP40 proteins. Considering the pronounced differences in transcriptional responses to MARV and SUDV in ERB-derived bmMΦs shown here, we explored whether the two viruses potentially exert differing IFN antagonistic properties, which could be associated with the distinct intracellular replication and viral progeny dynamics in these cells. For this, we used Spearman’s correlation analysis of the normalized gene counts of DEGs encoding key pattern-recognition receptor genes (*RIG-I* and *LGP2*), type I and type III IFNs (*IFNAs, IFNWs* and *IFNL*s), IFN receptors (*IFNAR1/2, IFNLR1* and *IL10RB*), transcriptional factors (*IRF1/3/4/7* and *NFKB1*) and interferon-stimulated genes (ISGs) (*ISG15/20*) against the two major antagonistic proteins of MARV (MARV-VP35 and MARV-VP40) and SUDV (SUDV-VP35 and SUDV-VP24), known to interfere with host IFN production, IFN-induced STAT signaling and phosphorylation, as well as RIG-I signaling.

**Figure 5:**
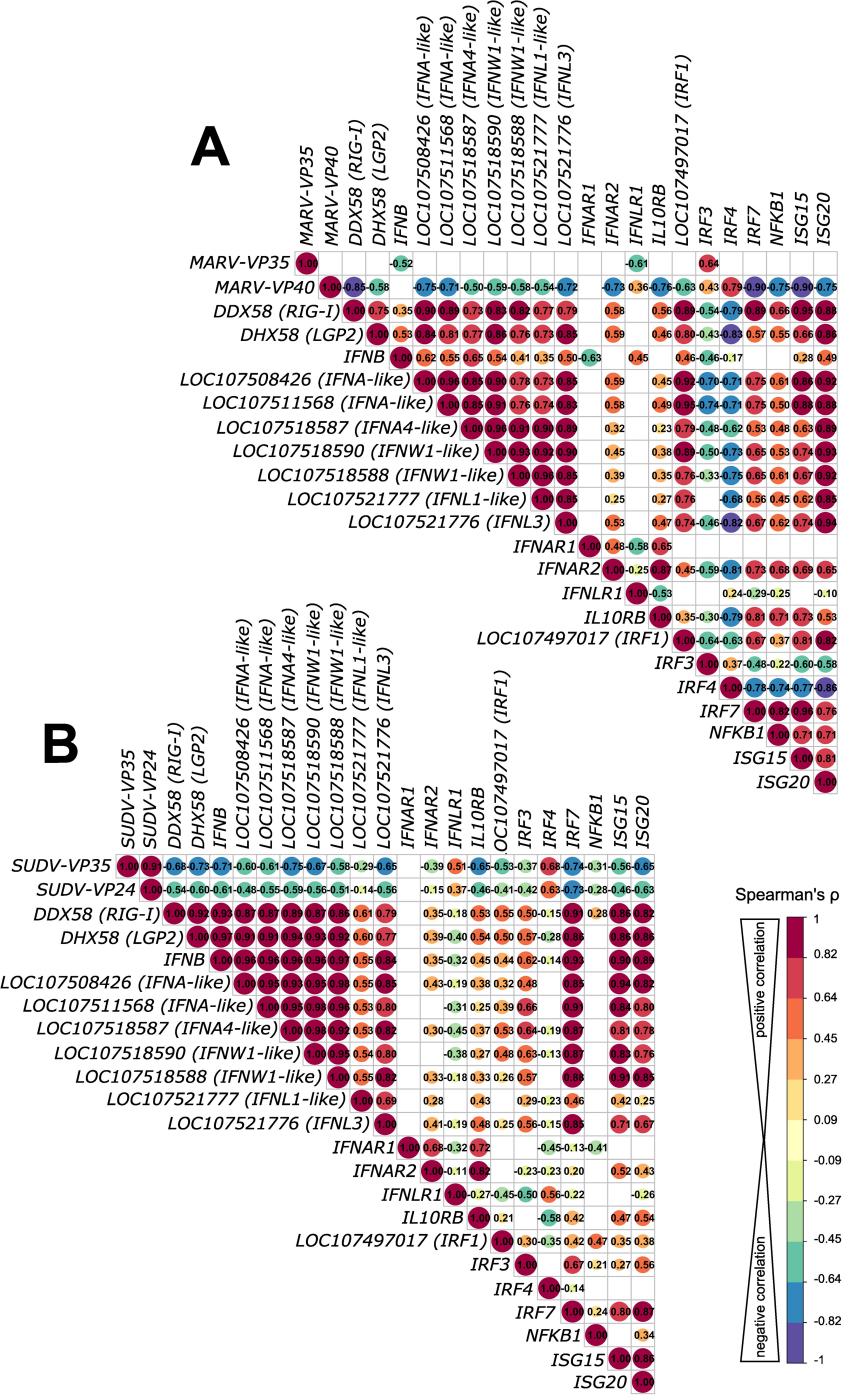
Correlation analysis of antagonistic filovirus gene and host antiviral gene expression patterns in bat bmMΦs. **(A)** Correlation matrix comparing correlations of gene expression of MARV infection-induced IFN response DEGs with MARV-VP35 and MARV-VP40. **(B)** Correlation matrix comparing correlations of gene expression of SUDV infection-induced IFN response DEGs with SUDV-VP35 and SUDV-VP24. The correlation matrix analysis used normalized gene counts of each gene as input. The data were analyzed using Spearman’s ρ correlation test using the corrr RStudio package. A perfect positive correlation is considered as having a ρ=1 and a perfect negative correlation a ρ=-1. Statistically insignificant correlations (*p*>0.05) are masked in the correlation matrices as empty boxes. The data used for the correlation analysis are pooled from four individual bats at 1, 2 and 3 DPI with each respective filovirus.

Correlation analysis revealed that MARV-VP35 gene expression showed a moderate negative correlation with only two of the 21 surveyed ERB IFN-associated genes – *IFNB1* (ρ=-0.52) and the type III IFN receptor *IFNLR1* (ρ=-0.61), indicative of severely limited VP35-driven suppression (**Figure 5A**). In stark contrast, SUDV-VP35 demonstrated a moderate to strong negative correlation with all but three surveyed IFN signaling-associated genes included in the analysis, suggestive of strong SUDV-VP35-driven antagonism of ERB IFN responses (**Figure 5B**). Unlike the negative correlation observed between MARV-VP35 and *IFNLR1*, SUDV-VP35 showed a moderate positive correlation with *IFNLR1*, in line with our earlier observation of differential induction of type III IFNs by SUDV but not MARV. Beyond the lack of negative correlation between MARV-VP35 and ERB IFN-associated genes, however, we found that MARV-VP40 exhibited stronger negative correlation with *DDX58* (RIG-I), two IFNA-like genes, *IFNL3, IFNAR2, IL10RB, IRF7, NFKB1, ISG15* and *ISG20* than either SUDV-VP35 or SUDV-VP24, indicative of strong MARV-VP40 antagonism and hence of major differences in how each virus potentially blocks or counteracts macrophage IFN responses in ERBs (**Figures 5A, B**).

### 2.6 MARV and SUDV elicit distinct signaling pathways in bat bmMΦs

**Figure 6:**
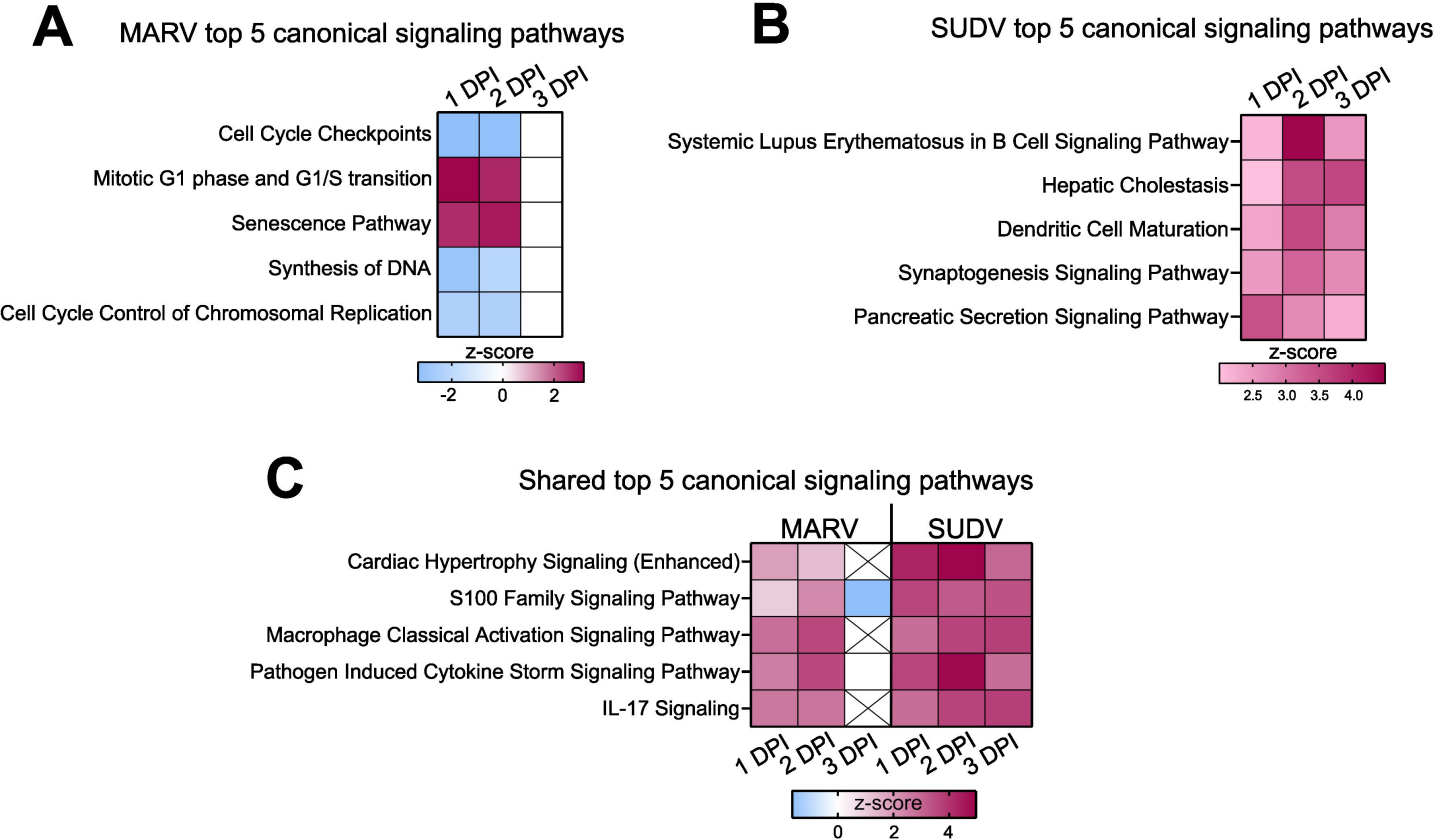
Differential regulation of canonical signaling pathways in filovirus-infected ERB bmMΦs. Ingenuity Pathway Analysis (IPA) of the top 5 statistically significant (*p* < 0.05) canonical signaling pathways in response to **(A)** MARV, **(B)** SUDV and **(C)** shared by both viruses at 1, 2 and 3 DPI, illustrated as z-score heatmaps.

Using Ingenuity Pathway Analysis (IPA), next we predicted what canonical signaling pathways are differentially regulated by the two filoviruses in bat-derived bmMΦs. Supplying the observed differential gene expression values for each virus at each day post-infection, the IPA software simulates the directional consequences of downstream molecules, infers upstream activity within given signaling pathways and predicts what upstream regulators may be causing observed gene expression changes and whether any canonical signaling pathways or biological processes are differentially regulated. Among the top 5 canonical signaling pathways regulated uniquely by MARV, we found two upregulated (*Mitotic G1 phase* and *G1/S transition and Senescence*) and three downregulated (*Cell Cycle Checkpoints, Synthesis of DNA* and *Cell Cycle Control of Chromosomal Replication*) pathways (**Figure 6A**). In contrast, the top 5 canonical pathways regulated uniquely by SUDV comprised only upregulated pathways, including *Systemic Lupus Erythematosus in B cell Signaling Pathway, Hepatic Cholestasis, Dendritic Cell Maturation, Synaptogenesis Signaling Pathway* and *Pancreatic Secretion Signaling Pathway* (**Figure 6B**). Among the top 5 shared canonical pathways regulated by both viruses were *Cardiac Hypertrophy Signaling (Enhanced), S100 Family Signaling Pathway, Macrophage Classical Activation Signaling Pathway, Pathogen Induced Cytokine Storm Signaling Pathway* and *IL-17 Signaling*, all of which were either downregulated or not at all regulated in MARV-infected cells by 3 DPI, but remained strongly upregulated in response to SUDV at all three timepoints (**Figure 6C**).

Next, we predicted what upstream regulators, molecules capable of regulating the expression, transcription or phosphorylation of other molecules, were differentially regulated in response to either MARV or SUDV infection. The top 20 IPA-predicted upstream regulators positively activated in response to MARV included various innate immune and proinflammatory response genes such as *TNF, IL1B, IFNG, CD40LG, TLR3* as well as *RELA*, an NFκB signaling-associated transcriptional factor. This response was largely limited to 1 DPI and 2 DPI for MARV, while at 3 DPI the top positively regulated upstream regulators only included *TNF* and several growth factors (*HGF, VEGF* and *EGF*) (**Supplementary Figure S4A**). In contrast, *IL10* encoding the canonical anti-inflammatory cytokine was negatively regulated consistently at all three timepoints in MARV-infected cells. At 3 DPI, MARV infection additionally resulted in the parallel inhibition of both *IL21* and *IL6*, two genes encoding a key immune regulatory cytokine and a canonical proinflammatory cytokine, respectively, indicating the orchestration of a carefully balanced immune response to MARV (**Supplementary Figure S4A**).

For SUDV, the top 20 positively regulated IPA-predicted upstream regulators included a mix of innate immune genes, transcriptional and growth factors. Reflecting some of the findings for MARV, SUDV-induced upstream regulators included *TNF, IL1B, CD40LG, RELA, IFNG* and *TLR3*. However, additional upstream regulators were also positively regulated in response to SUDV, such as *IRF1, IRF7*, *poly rI:rC RNA* and *NFKB (complex)*, as well as growth factors *VEGF, NGF* and *EGF* (**Supplementary Figure S4B**).

### 2.7 Pathogen-induced responses in ERB bmMΦs suggest divergent regulation of downstream immune signaling

**Figure 7:**
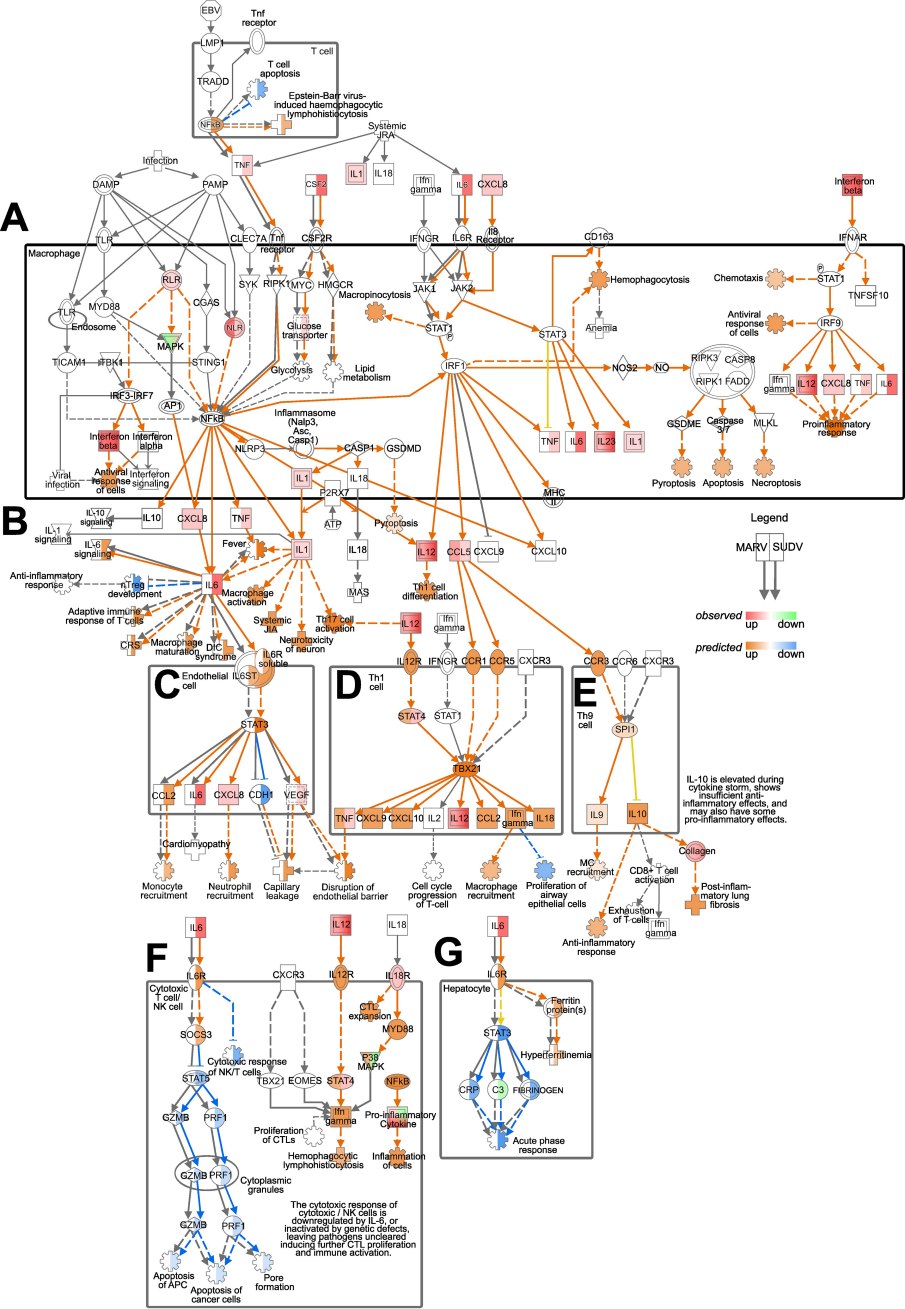
Pathogen Induced Cytokine Storm Signaling Pathway regulation by MARV and SUDV in ERBs. The map was generated using the IPA molecular activity predictor (MAP) analysis of the canonical Pathogen Induced Cytokine Storm Signaling Pathway **(A)** in macrophages, **(B)** exogenously, **(C)** in endothelial cells, **(D)** in Th1 cells, **(E)** in Th9 cells, **(F)** in cytotoxic T cells/NK cells and **(G)** in hepatocytes. Within macrophages and for exogenous gene expression **(A, B)**, only genes actually observed in our data as differentially expressed are indicated as red (upregulated) or green (downregulated), while downstream responses (cartwheel and cross symbols) predicted as differentially regulated (orange for upregulated and blue for downregulated) based on the true DEGs are included. For the downstream signaling and responses in other cell types in this pathway **(C–G)** both real observed (red-green) and predicted (orange-blue) gene expression and downstream processes are included. For every symbol, the left half always corresponds to the relevant response or prediction for MARV, and the right half corresponds to the relevant response or prediction for SUDV. Orange arrows represent predicted upregulation, blue arrows predicted downregulation, yellow arrows ambiguous prediction and grey arrows no differential regulation.

Considering the clear differences in transcriptional responses to MARV and SUDV in ERB bmMΦs, next we focused on surveying in more detail any discrepancies in the regulation of genes comprising the *Pathogen Induced Cytokine Storm Signaling Pathway*, predicted by IPA as differentially regulated by both viruses. Using IPA’s Molecular Activity Predictor (MAP) tool, we explored signaling cascades observed and predicted by MAP as differentially regulated in macrophages, endothelial cells, hepatocytes and various T cell subsets. We directly compared the gene expression regulation by MARV and SUDV, choosing the 2 DPI timepoint to survey the peak transcriptional response changes in responses to both viruses (**Figure 7**).

In T cells, the predicted upregulation of *NFκB* in response to SUDV was forecasted to suppress T cell apoptosis, contrasting the absence of predicted *NFκB* expression for MARV-infected cells. Within the macrophage compartment, the observed upregulated expression of *TNF, IL6* and *CSF2* in response to SUDV, but not MARV, reflected in the differential prediction for *RIPK1* and *NFkB* signaling, glucose transport, lipid metabolism, glycolysis and JAK1/JAK2 signaling between MARV and SUDV (**Figure 7A**). Despite the differential *TNF* and *IL6* responses, however, exogenous *IFNB* signaling and the upregulation of *IL12* and *CXCL8* in response to both viruses in macrophages resulted in similar predictions for upregulated chemotaxis, antiviral response of cells and proinflammatory response (**Figure 7A**). Exogenously, the upregulated expression of IL6 signaling by SUDV was predicted to induce diverse downstream processes, including adaptive immune response of T cells, macrophage activation and maturation, fever and the suppression of nTreg development, none of which were predicted as induced in MARV-infected cells. In contrast, the upregulation of *IL12* in response to both viruses was predicted to induce Th1 cell differentiation (**Figure 7B**).

The observed upregulated expression of *CXCL8, IL1, IL12* and *CCL5*, coupled with the divergent *TNF* and *IL6* responses were predicted to heavily influence the forecasted downstream signaling processes in diverse cell types. In endothelial cells, *IL6* signaling from SUDV-infected but not MARV-infected macrophages was predicted to induce *STAT3* expression, the upregulation of *CCL2* and the suppression of *CDH1*. Combined with the observed upregulation of *CXCL8* and *VEGF*, the predicted outcome for SUDV infection included increased monocyte and neutrophil recruitment, paralleled by capillary leakage and disruption of the endothelial barrier (**Figure 7C**). In Th1 and Th9 cells, on the other hand, *CCL5* signaling through the chemokine receptors CCR1, CCR5 and CCR3 was predicted to induce comparable upregulated expression of *STAT4, TBX21, CXCL9, CXCL10, CCL2, IFNγ* and *IL18* in response to both viruses. Downstream macrophage and mast cell (MC) recruitment, the suppression of proliferation of airway epithelial cells, anti-inflammatory response and post-inflammatory lung fibrosis were also predicted as similarly regulated in MARV and SUDV-infected cells (**Figures 7D, E**).

A strong and clear difference in cytokine signaling between MARV and SUDV was also predicted in cytotoxic T cells/NK cells and hepatocytes. In cytotoxic T cells/NK cells, we found that SUDV-induced *IL-6* was predicted to induce *SOCS3* expression and the downstream suppression of *STAT5*, granzyme B (*GZMB*) and *PRF1* expression, leading to suppressed apoptosis of APCs and cancer cells, as well as pore formation (**Figure 7F**). In hepatocytes, on the other hand, the presence of *IL6* signaling in SUDV-infected cells was predicted to induce hyperferritinemia, the suppression of *STAT3*, C-reactive protein (*CRP*), fibrinogen expression and the downregulation of acute phase response (**Figure 7G**). Due to the absence of *IL6* signaling in MARV-infected cells, these genes and pathways remained blank in the MAP analysis, indicating the absence of MARV-induced regulation along these signaling cascades in cytotoxic T cells/NK cells and hepatocytes (**Figure 7G**).

### 2.8 ERB bmMΦs display a distinct response to MARV compared with human MΦs

**Figure 8:**
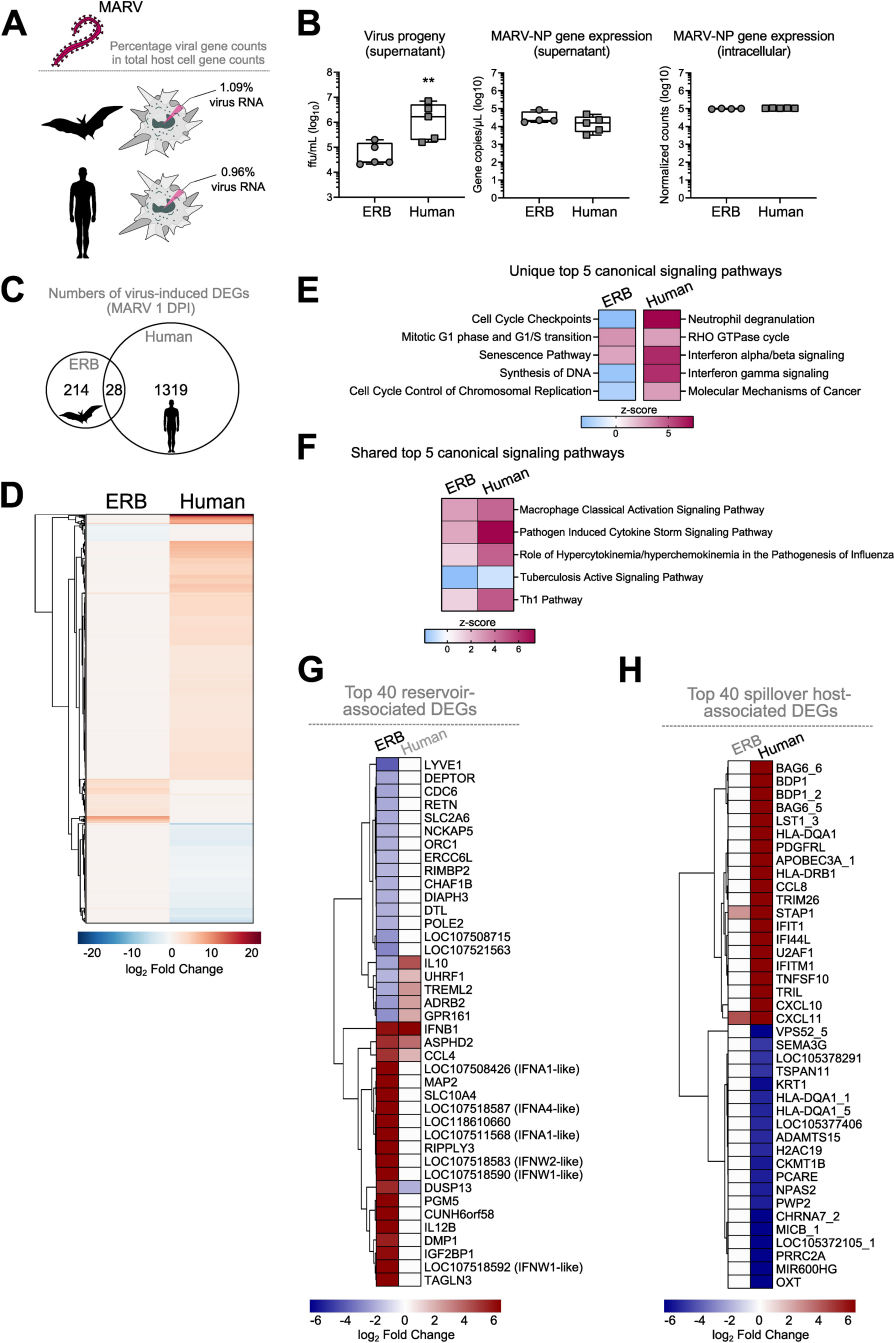
Comparative DEG profile of ERB-derived bmMΦs and human monocyte-derived MΦs (mo MΦs) at 1 DPI with MARV. **(A)** Percentages of viral RNA within total RNA counts in ERB and human MΦs. The percentage was calculated using the sum of normalized gene counts of all 7 MARV genes against the sum of all host gene counts within the bulk RNAseq dataset for each species. **(B)** Viral replication represented as virus progeny in cell culture supernatants (left), MARV-NP gene counts in cell culture supernatants (middle) and intracellular MARV-NP gene counts (right). **(C)** A Venn diagram illustrating the total numbers of unique and shared DEGs induced by MARV in ERB bmMΦs and human moMΦs at 1 DPI. **(D)** Global heatmap of DEG expression in MARV-infected ERB bmMΦs and human moMΦs at 1 DPI. IPA of the top 5 statistically significant (*p* < 0.05) canonical signaling pathways at 1 DPI with MARV **(E)** unique to either ERB or human MΦs and **(F)** shared by ERB and human MΦs, illustrated as z-score heatmaps. **(G)** A heatmap of the top 20 upregulated and top 20 downregulated genes induced in MARV-infected ERB bmMΦs, plotted against the expression of their human orthologs. **(H)** A heatmap of the top 20 upregulated and top 20 downregulated genes induced in MARV-infected human moMΦs, plotted against the expression of their ERB orthologs. DEGs for each species were defined as genes with a *p-adj* < 0.05 and a log_2_-fold change ≥ ± 1.5. The ERB dataset includes pooled data from four individual bats, while the human dataset includes pooled data from three individual healthy donors. The heatmaps are shown as log_2_-fold change against respective mock-infected negative controls for each species. Statistical analysis in **(B)** was performed using a Mann-Whitney-U-test. **p < 0.01.

Finally, we compared the transcriptional responses of ERB MΦs observed herein against our recently published dataset of human monocyte-derived MΦ (moMΦ) responses to MARV to directly relate any similarities or differences in cell responses in the natural reservoir versus the spillover host (36). For this, we first quantified and compared the intracellular viral replication in ERB and human moMΦs by calculating the percentage of the total gene counts that constituted viral genes in each species. We found very similar intracellular MARV RNA loads in each host, with viral gene counts constituting 1.09% of total gene counts in ERB bmMΦs and 0.96% in human moMΦs (**Figures 8A, B**). We then compared viral loads in cell culture supernatants and found that despite similar MARV-NP gene copies intracellularly and in cell culture supernatants, human moMΦs released significantly more infectious viral particles than ERB bmMΦs (**Figure 8B**).

The early transcriptional response to MARV included the differential expression of 1319 genes in human moMΦs, contrasted by 214 genes induced in ERBs and only 28 shared DEGs induced in both species, highlighting significant qualitative and quantitative differences in bat and human macrophage responses to MARV *in vitro* (**Figures 8C, D**). IPA of the top 5 canonical signaling pathways induced uniquely by ERB or human macrophages also revealed that, unlike the signaling pathway profiles in ERBs described for MARV and SUDV, human moMΦs significantly upregulated distinct signaling pathways such as *Neutrophil degranulation, RHO GTPase cycle, Interferon alpha/beta signaling, Interferon gamma signaling* and *Molecular Mechanisms of Cancer* (**Figure 8E**). In contrast, among the top 5 canonical signaling pathways regulated commonly by both ERB and human MΦs infected with MARV were *Macrophage Classical Activation Signaling Pathway, Pathogen Induced Cytokine Storm Signaling Pathway, Role of Hypercytokinemia/hyperchemokinemia in the pathogenesis of Influenza*, *Tuberculosis Active Signaling Pathway* and *Th1 Pathway* (**Figure 8F**).

To explore in greater detail the MΦ transcriptional profile in each species, next we extracted the top 20 up- and top 20 down-regulated genes of ERBs and humans, designating each gene set as the “Top 40 reservoir-associated DEGs” and “Top 40 spillover host-associated DEGs”, respectively (**Figures 8G, H**). We observed very limited overlap in antiviral response gene expression between ERBs and humans, with only *ASPHD2*, *IFNB1* and *CCL4* being similarly upregulated in both hosts. The dual specificity phosphatase gene *DUSP13* was upregulated in ERBs, but downregulated in humans, while five other genes were downregulated in ERBs but upregulated in human moMΦs (*IL10, UHRF1, TREML2, ADRB2, GPR161*) (**Figure 8G**). Interestingly, *MAP2, RIPPLY3* and *TAGLN3* shown earlier to be upregulated by MARV and not SUDV in ERBs were not induced in MARV-infected human moMΦs either, further indicating a specific role for these cytoskeleton and polymerase-associated genes restricted to the ERB-MARV context (**Figures 4D** **,** **8G**). In contrast, within the top 40 spillover host-associated DEGs, only two genes were mutually upregulated in both ERBs and humans – the chemokine *CXCL11* and the signal transduction adaptor protein gene *STAP1* (**Figure 8H**). Unlike ERBs, human moMΦs also displayed simultaneous upregulation and downregulation of various HLA genes (*HLA-DQA1s* and *HLA-DRB1*), cytokines and chemokines (*CCL8, CXCL10, CXCL11, TNFSF10*), ISGs (*IFIT1, IFI44L, IFTM1*) and various antiviral-associated genes (*APOBEC3A, TRIM26*), classical signs of filovirus-induced deregulation of host macrophage functionality (**Figure 8H**).

## 3 Discussion

ERBs have been extensively established as natural reservoirs of MARV and while the reservoirs of pathogenic orthoebolaviruses like EBOV and SUDV are yet to be discovered, several bat species are considered credible candidates (18, 19, 39). ERBs represent unlikely orthoebolavirus reservoirs, as they’re largely refractory to experimental infections with EBOV, BDBV, TAFV or RESTV (26, 40). However, SUDV is capable of limited tissue-restricted replication in ERB liver, spleen and axillary lymph nodes, offering an invaluable opportunity to explore what features of the ERB innate immune response are modulated through co-adaptation with MARV, in contrast with protective immunity to SUDV infection. More specifically, we sought to compare MΦ transcriptional responses to MARV and SUDV with the assumption that while MARV will induce unique transcriptional regulation driven by co-evolution with ERBs, SUDV should induce non-adapted responses resembling those of a foreign viral infection. This would allow us to elucidate some of the augmented features of the reservoir-virus response that allow for population-level maintenance of MARV, which likely accommodate sufficient MARV replication in an ERB to facilitate onward virus transmission to other bats, as opposed to being cleared within days with no significant shedding like that seen in SUDV-infected ERBs. We chose MΦs as they represent an important early host target of filoviruses in both humans and bats, their dysregulation being a central factor in primate filovirus disease progression and immunopathology.

Gaining a deeper understanding of the molecular and cellular mechanisms underlying the zoonotic reservoir competence of bats has been confounded by a distinct paucity of species-specific laboratory reagents and assays. Recent in-depth analyses of bat genomes have revealed new insights into numerous evolutionary adaptations of chiropteran immune systems that some viruses have likely leveraged as platforms for co-adaptation, contributing to the zoonotic reservoir status of some bats. Various bat species appear to have acquired expanded natural killer cell receptor genes (41) or alternatively have undergone gene loss of *α* and *β defensin* genes (42), killer cell lectin-like receptor K1 (*KLRK1*), PYRIN and HIN domain (*PYHIN*) genes (43, 44) or proinflammatory cytokine genes like *IL36A* and *IL36G* (9). Others have experienced significant positive selection of viral sensors and inflammatory response regulators like *TLR8* and *TRIM38* or the deletion of function-altering Cys residues in IFN-associated genes like *ISG15* (9). Unique patterns of IRF7-driven induction of ISGs in black flying foxes (*P. alecto*) were also recently reported (43). Combined, these findings illustrate that different bats have evolved diverse and highly-specific molecular mechanisms that likely contribute to their ability to tolerate, maintain and transmit some viral pathogens by striking a fine-tuned balance between the induction of sufficient antiviral immune responses and the absence of aberrant tissue-damaging inflammatory processes.

Studies have previously reported the successful *in vitro* differentiation and characterization of bat bmMΦs from black flying foxes (*P. alecto*) and cave nectar bats (*Eonycteris* sp*elaea*) (44, 45). However, few studies have focused on developing MΦ cultures from known bat reservoirs of zoonotic diseases. Taking advantage of limited bat resources, we successfully differentiated ERB-derived bmMΦs for an immunological interrogation of filovirus-specific *in vitro* responses. In line with the reservoir competence of ERBs, their bmMΦs were readily susceptible to MARV and maintained stable low-level virus replication over the course of 3 days, similar to ERB-derived bmDCs (28). Moreover, the previously reported transcriptional profile of MARV-infected bmDCs reflected in our current observations of the upregulation of only a restricted set of cytokines, chemokines, type I IFNs, ISGs and transcriptional factors in MARV-infected bmMΦs mostly restricted to 1 DPI (28). For SUDV we detected notably higher intracellular viral loads than for MARV at the same MOI, coupled with declining viral production in cell culture supernatants, mirroring the *in vivo* differential control kinetics of MARV and SUDV reported following experimental infections of ERBs (26).

Regardless of the immune or non-immune origin of ERB cells (bmMΦ *vs*. RoNi), MARV infected fewer cells and replicated less than SUDV over a 3-day *in vitro* infection, contrasting comparable replication rates of MARV and SUDV in Vero E6 cultures. Moreover, despite similar intracellular replication in bat and human MΦs, MARV-infected ERB bmMΦ cultures contained significantly fewer infectious viral particles than human moMΦs, indicating the presence of host-specific intrinsic differences in viral replication processes unique to the ERB-MARV relationship.

Viruses have evolved to rely on, exploit and remodel the host cell machinery for their entry, replication, trafficking, shuttling and virion egress (46). As a result, they also inadvertently remodel the host cell actin cytoskeleton and can even prime RIG-I-like receptor activation in response to cytoskeleton disturbances (47). The directed transport of viral proteins typically takes place alongside cytoskeletal tracks like microtubules or actin filaments (48–50). Here, we found that the top DEGs induced by MARV in ERB bmMΦs included several genes encoding microtubule-associated proteins (MAPs) and two genes associated with RNA polymerase activity. Importantly, neither SUDV-infected bat bmMΦs, nor MARV-infected human moMΦs induced the expression of any of these genes, highlighting the presence of host cell cytoskeleton and polymerase activity alterations unique to MARV-ERB infection settings. Prior evidence of filovirus protein association with host cell microtubules has shown an association of EBOV matrix protein VP40 with host cell microtubules, resulting in the stabilization of cellular microtubules against drug-induced depolymerization and enhancement of tubulin polymerization - properties similar to those of MAPs (46). In contrast, drug-induced depolymerization of microtubules in a human macrophage cell line, but not in other cell lines, increases MARV viral protein release *in vitro*, indicating a cell type-specific association between MARV viral progeny release and host cell microtubule organization (51). Based on these and our own findings, we therefore hypothesize that the ability of MARV to maintain low-level productive infections in these bats could be aided by viral co-adaptations that manipulate the intracellular replication machinery of ERBs to preserve viral replication rates sufficient to maintain viral transmission, but low enough to avoid inducing overt immune responses and rapid viral clearance.

Despite the stable MARV replication rates at 1, 2 and 3 DPI, ERB bmMΦs underwent an almost complete transcriptional shut-down by 3 DPI, contrasting the stronger and more diverse transcriptional changes maintained in SUDV-infected cells at the same timepoints. This striking phenomenon was evident both when looking at immune-related DEG expression and in our IPA dataset, which highlighted the presence of MARV-induced suppression of canonical signaling pathways associated with cell cycle control, DNA synthesis, mitosis and cell senescence. Together, these findings suggest the presence of expansive transcriptional silencing of a wide range of cellular processes in MARV-infected ERB bmMΦs by 3 DPI. Moreover, even though MARV replicated at similar rates in bat and human MΦs, we observed stronger and more diverse transcriptional responses to MARV in human MΦs than ERB bmMΦs. Viral replication dynamics alone are therefore an unlikely driver of the magnitude and nature of the transcriptional response profile of filovirus-infected host cells described herein. These responses likely rely on a combination of complex factors involving species-specific virus-driven immune modulation and antagonism, and in the case of ERBs and MARV, the accumulation of numerous co-evolutionary adaptations in both the reservoir and virus.

The exceptionally high virulence of filoviruses in humans and NHPs is at least partially explained by the potent immune inhibitory properties of several virus-encoded proteins. Each filovirus employs its own strategies interfering with host antiviral immune responses. EBOV-VP24 inhibits IFN-induced JAK-STAT signaling (33), while MARV-VP40 blocks STAT protein tyrosine phosphorylation (52). EBOV-VP35 and MARV-VP35 both block IFN production by interfering with RIG-I signaling, albeit through different dsRNA interaction mechanisms and with different efficiencies (53–58). Mutating specific MARV-VP35 residues associated with dsRNA binding results in improved type I IFN responses and reduced viral replication, demonstrating a central role of VP35 as a virulence factor (53, 54, 59–61). Herein, we found stark differences in MARV-VP35 and SUDV-VP35 correlation with several bat pattern recognition receptors (PRRs), type I and III IFNs and ISGs. MARV-VP35 showed severely limited negative correlation with any of the tested DEGs, while SUDV-VP35 displayed moderate to strong negative correlation with most IFN-associated genes, indicating intact IFN antagonistic properties of SUDV-VP35. Contrasting MARV-VP35, MARV-VP40 showed a markedly stronger negative correlation with *RIG-I*, type I and III IFNs, IFN receptors *IFNAR2* and *IL10RB*, *IRF7*, *NFKB1, ISG15* and *ISG20* than either SUDV-VP24 or SUDV-VP40. Considering the largely muted transcriptional changes observed in MARV-infected bmMΦs by 3 DPI, it is therefore conceivable that MARV-VP40 has likely co-evolved selective and unique IFN antagonistic properties that target ERB antiviral responses more efficiently than other filoviruses and contribute to the ability of these bats to maintain low-level MARV infections in the general absence of significant IFN-driven responses and rapid viral clearance. In contrast, our findings suggested that MARV-VP35 possibly displays reduced antagonistic abilities in these bats.

The type I (α, β, δ, ω and ε) and type III IFN families (λ) represent key components of the early host antiviral immune response. The initial recognition of viral RNA by host PRRs induces the production of IFNs, which bind and signal through the heterodimeric receptor complex IFNAR1/2 (type I IFN) or IFNLR1/IL10RB (type III IFN) and trigger the downstream expression of diverse ISGs and in the case of IFNλs, also the induction of B- and T-cell driven adaptive immune responses (62–67). Studies have now identified various noteworthy differences in the IFN repertoires of humans and bats, such as a contracted type I IFN locus in black flying foxes (*P. alecto*) or an expansion of type I IFN loci in large flying foxes (*P. vampyrus*) and little brown bats (*Myotis lucifugus*) (68, 69). In ERBs, the IFN-ω gene family has undergone considerable expansion and contains 22 *IFNW* genes, contrasting with only 5 and 6 *IFNW* genes in *P. alecto* and *Rhinolophus ferrumequinum* bats, respectively, and a single *IFNW* gene in humans (41, 70). Even though *IFNW* genes do not show constitutive expression in ERBs, SeV infection induces upregulated transcript levels in immortalized RoNi cells (41). Moreover, recombinant IFN-ω4 can block experimental infection with a recombinant VSV in the same cell line, demonstrating measurable antiviral activity of ERB-derived IFN-ω (41). Herein, we found that infection with MARV and SUDV induced an overall similar pattern of upregulated type I IFN expression. However, MARV failed to induce type III IFN transcription in bmMΦs, while SUDV triggered the strong upregulated expression of two type III IFN genes (*IFNL1-like* and *IFNL3)* at 2 and 3 DPI. Interestingly, ERB-derived lung organoids undergo significant upregulation of both the *IFN1-like* and *IFNL3* genes at 3 days post-infection with MARV, as well as in response to SeV, H1N1 influenza virus and VSV infections. Moreover, this type III IFN response in bat organoids appears to drive robust, protective and self-amplified antiviral responses (71). Considering the strong negative correlation between MARV-VP40, *IL10RB, IFNL1-like, IFNL3*, various transcriptional factors and ISGs, we therefore hypothesize that MARV has evolved unique VP40 antagonistic properties that specifically target ERB type III IFN responses, subduing IFNλ production, downstream signaling and adaptive immune response activation. Additionally, given the key role of macrophages as both early targets of filoviruses and coordinators of innate and adaptive host responses, the absence of type III IFN responses in ERB-derived MΦs could be a cell-specific response aimed at subduing downstream activation of further adaptive immune responses to MARV. In contrast, the weaker SUDV-VP24 and SUDV-VP35 negative correlation with these genes indicates that unlike MARV, SUDV infection induces sufficient type III IFN responses in ERBs to potentially contribute to increased T and B cell proliferation and the ability of these bats to rapidly control SUDV infections *in vivo*.

To test whether the overall pathogen response observed in ERB bmMΦs potentially translates in differential adaptive immune responses to filoviruses, we performed a comprehensive IPA analysis of the canonical *Pathogen Induced Cytokine Storm Signaling Pathway*, regulated by both MARV and SUDV in these cells, as well as in human moMΦs. We found that the absence of *TNF* and *IL6* signaling in MARV-infected cells and the significant upregulation of both cytokines in SUDV-infected cells lead to significant disparities in downstream immune cell activation and regulation. In response to SUDV, *TNF* and *IL6* signaling were predicted to suppress T cell and APC apoptosis, Treg development, pore formation and acute phase response in hepatocytes. The *TNF* and *IL6* signaling cascades were predicted to induce adaptive immune responses of T cells, the recruitment of various innate immune cells, including monocytes, macrophages, neutrophils and mast cells, as well as both antiviral responses and anti-inflammatory responses. Combined with the induction of type III IFNs by SUDV and not MARV, our findings therefore highlight starkly different responses of ERBs to each virus and point to the induction of an unperturbed and well-balanced proinflammatory response to SUDV, paralleled by downstream recruitment and activation of endothelial cells, hepatocytes and T cells that likely contribute to the previously reported clearance of SUDV infection *in vivo* (26). In contrast, the absence of *TNF, IL6* and type III IFN responses to MARV in these bmMΦs are likely contributing factors to the muted transcriptional response to MARV in these cells. Unlike ERBs, elevated TNF, IL-6 and IL-10 cytokine responses are classical hallmarks of severe filovirus disease in both humans and NHPs following natural exposure or experimental infections (72–76). Exposing human or NHP peripheral blood mononuclear cells (PBMCs) to filoviral peptides or inactivated viral particles also results in cell apoptosis, inhibition of CD4 and CD8 T cell cycle and maturation, and increased IL-10 production, resulting in an overall dysfunctional T cell response and the development of severe tissue pathology (77). This pronounced immune dysfunction appears to be long-lasting, with EBOV survivors displaying elevated blood markers of inflammation, including high levels of IL-1β, TNF and CCL5, increased anti-inflammatory IL-10, sustained T cell activation and DC depletion 19–25 months post-infection, none of which are evident in filovirus-infected ERB bmMΦs (78).

Additional work in our group recently offered a comprehensive comparative peripheral blood response analysis of ERB and NHP/human responses to MARV, EBOV and SUDV and highlighted remarkable consistency in transcriptional responses to all three viruses across primate studies (79). Despite marked differences in experimental set-ups between these studies, a core set of canonical genes typically associated with mammalian antiviral responses and pathogenesis were evident in humans, NHPs and bats (79). Those included key PRRs (*IFIH1/MDA5* and *DDX58/RIG-I*) and antiviral genes (eg. *ISG15, ISG20, IRF7, MX1, OAS1, OAS3, IFITs, STAT1, STAT2, FOS*). We also described clear divergent peripheral immune responses to filoviruses between bats and primates, the latter significantly upregulating various recognition receptors (eg. *TLR3, TLR4, DHX58*), antiviral genes (eg. *IFI35, OAS2, MX2*), proinflammatory cytokine and chemokine genes (eg. *CXCL10, IL6, CCL2/3/8, CXCL11, IL1B*), in line with the highly activated proinflammatory transcriptional profile of MARV-infected human macrophages (36, 79). As natural reservoirs, ERBs likely hold a number of critical evolutionary advantages in modulating MARV infection, replication and transmission over primate spillover hosts, whilst remaining asymptomatic and avoiding overt proinflammatory processes.

Herein, we found that beyond bulk blood cell or tissue-level responses, ERBs employ carefully fine-tuned pathogen-specific responses to different filoviruses at specific innate immune cell levels. Bat bmMΦ responses were characterized by a muted antiviral response to MARV, contrasted by a stronger, sustained and proinflammatory-skewed response to SUDV reminiscent of the strong filovirus-induced responses in humans and NHPs. Despite the presence of limited shared gene signatures between MARV and SUDV responses in ERBs, and even fewer similarities between bat and human responses to MARV, we identify several molecular mechanisms differentially regulated in these bats. We show evidence of virus-specific host cell cytoskeletal changes, unique patterns of viral protein antagonism, type III IFN responses, as well as differential TNF and IL6 responses. The absence of these mechanisms in response to MARV are a possible result of the highly specific coevolutionary relationship between MARV and its natural wildlife reservoir, allowing these bats to maintain and transmit MARV at low levels without developing signs of viral hemorrhagic fever disease themselves. In contrast, their induction in response to other filoviruses is a likely contributing factor to the ability of ERBs to clear orthoebolaviruses like SUDV. Even though these bats control MARV infections in the wild, they do allow for sufficient viral replication and persistence to maintain and transmit MARV at the population level. Thus, based on our findings, MARV has likely co-adapted to ERBs in a way that tempers innate immune responses enough to allow low-level viral replication sufficient for transmission in the absence of aberrant innate immune cell activation, but also without interfering with the generation of protective T and B cell responses.

## 4 Materials and methods

### 4.1 *In vitro* differentiation of ERB-derived bmMΦs

Bone marrow cells were obtained from captive ERBs euthanized for unrelated studies at the CDC with prior approval from the Centers for Disease Control and Prevention Institutional Animal Care and Use Committee and in strict accordance with the Guide for the Care and Use of Laboratory animals and following cell isolation protocols as previously described (15, 28). For the current study, no live animal work, no anesthesia or euthanasia were necessary and all work performed herein involved only *in vitro* cell culture techniques using bone marrow cells obtained from past studies (15, 28). To differentiate bmMΦs, one vial of cryopreserved bone marrow cells per bat was thawed from a total of four bats and resuspended in 9 mL of R10 medium containing 10% FCS, 1% L-glutamine, 1% penicillin and streptomycin, 1% HEPES and benzonase (10 µL/100mL medium) in RPMI-1640 medium (Sigma). The cell suspension was centrifuged at 350x g for 10 minutes and the R10 wash medium was carefully removed. The cell pellet was slowly resuspended in fresh R10 medium containing 20 ng/mL recombinant ERB macrophage colony-stimulating factor (M-CSF, Kingfisher Biotech). The cells were then plated out at an approximate density of 5x10^5^ cells/well in a final volume of 250 µL and incubated at 37°C in 5% CO_2_. After one day of incubation, the cells were supplemented with 250 µL/well of fresh pre-warmed R10+M-CSF medium. The medium was added slowly and drop-wise to the center of each well. The plate was then returned to the incubator. On days 3 and 6, half of the medium in each well was carefully removed and was replaced with 250 µL of fresh pre-warmed R10+M-CSF medium as described above.

On day 8 of differentiation, the cell culture medium was carefully removed, and adherent cells were gently washed by slowly adding 500 µL/well of pre-warmed PBS and carefully removing it again. At this stage, cell density and morphology were controlled visually under a microscope. One well of cells per bat on each plate was always designated for cell dissociation and counting prior to restimulation or infection.

### 4.2 Cell stimulation and virus infections

Prior to infection or restimulation, the culture medium in the wells containing bmMΦs designated for cell counting was carefully removed. The cells were washed with 500 µL/well of pre-warmed PBS as described above and Cell Dissociation Buffer (Life Technologies Corporation) was added to each well. In brief, for cell dissociation, 500 µL of Cell Dissociation buffer was added to each well, and cells were incubated at room temperature for 15 min, occasionally tapping and swirling the plate to facilitate cell detachment from the plastic. After 15 min, the cells were then gently dissociated by repeated pipetting and scraping of each well with the pipette tip. The buffer containing the detached cells was transferred in fresh 2 mL centrifuge tubes. The wells were washed with 500 µL of PBS, which was then added to the cell suspension. The cells were centrifuged at 350x g for 5 minutes. The buffer was then carefully removed and the cells were resuspended in 500 µL of R10 medium. Dissociated cells were stained with trypan blue and were manually counted under a microscope using Neubauer chambers. The obtained cell counts were then used to calculate the appropriate volume of virus needed to achieve the desired multiplicity of infection (MOI).

For stimulation with bacteria lipopolysaccharide (LPS), bmMΦs from four individual bats were incubated in 250 µL of R10+M-CSF medium containing 2 µg/mL LPS (InvivoGen). For Sendai virus (SeV) infection, cells were incubated in 250 µL R10+M-CSF medium containing 30 hemagglutination (HA) units of the Cantell strain, a non-pathogenic paramyxovirus used as a positive stimulation control for host IFN signaling. For filovirus infections, bmMΦs were covered in 100 µL of R10+M-CSF medium to prevent desiccation and were infected with either MARV (isolate Uganda 200704852 Uganda Bat, MARV371), MARV expressing green fluorescent ZsG protein (MARV-ZsG), SUDV-Gulu or SUDV-Gulu expressing ZsG (SUDV-ZsG) at an MOI of 2 (as titrated on Vero E6 cells). Cells were incubated with each virus inoculum for 1 h at 37°C in 5% CO_2_ with gentle mixing every 15 min by slowly swirling the plate to ensure even inoculum distribution in each well. The virus inoculum was then carefully removed, the cells were washed in pre-warmed PBS and fresh 250 µL R10+M-CSF medium was slowly added to each filovirus-infected well. Mock-infected bmMΦs were cultured in R10+M-CSF medium only. At indicated timepoints, cell culture supernatants were collected for virus isolation, viral and cell RNA extraction, while bmMΦs were dissociated from the plates as described above for staining and flow cytometry.

Immortalized RoNi cells and Vero E6 cells were plated in triplicate in 24-well tissue culture-treated plates at an approximate density of 2x10^5^ cells/well in 1mL/well of DMEM medium containing 10% FCS, 1% L-glutamine and 1% Penicillin/Streptomycin. In the BSL-4 lab, the medium was carefully removed using a multi-channel pipette and the cells were washed once using fresh pre-warmed DMEM medium. Each cell type was then infected with either MARV-ZsG or SUDV-ZsG at an MOI of 2. Mock-infected cells were included as controls. The protocol for cell infection, incubation, cell dissociation and staining for flow cytometry was as described above for ERB bmMΦs.

Work with wild-type and recombinant ZsG filoviruses was conducted at the Robert Koch Institute under Biosafety Level 4 (BSL4) laboratory conditions. Research staff involved in this study adhered closely to all approved BSL4 safety protocols and standard operating procedures (SOPs) for sample inactivation and removal from the BSL4 facility.

### 4.3 Flow cytometry

For flow cytometry analysis of bmMΦ surface marker expression, mock-infected or filovirus-infected cells were harvested as described above. Cells were transferred in 2 mL tubes and were stained in 30 µL per sample of antibody mix in FACS buffer (protein-free PBS containing 0.2% BSA and 2 mM EDTA) with Live/Dead Fixable Yellow Dead Cell Stain Kit (Invitrogen) and antibodies raised against the following markers: anti-mouse CD11b-PE (clone M1/70 diluted 1:100, BD), anti-human HLA-DR-A785 (clone L243 diluted 1:50, BioLegend), anti-human CD40-PE-Cy7 (clone 5C3 diluted 1:20, BioLegend), anti-human CD163-AF674 (clone QA19A16 diluted 1:100, BioLegend) and anti-human CD206-PB (clone 15–2 diluted 1:100, BioLegend). A custom-made anti-bat CD14 antibody conjugated in-house with either PerCP or AF647 LightningLink kits (Abcam) as per the manufacturer’s instructions was also included in the staining panel (CDC, diluted 1:100). Cells were stained for 15 min at room temperature and were then washed once in 200 µL/sample of FACS buffer. Stained and washed cells were fixed overnight in 200 µL/sample of 10% formalin. Following overnight fixation, cells were transferred in fresh 200 µL formalin and were removed from the BSL4 laboratory in accordance with approved SOPs. Samples were run on a Cytoflex S cytometer (Beckman Coulter GmbH) and the final results were analyzed using FlowJo software version 10.8.1 (TreeStar).

### 4.4 Confocal microscopy

For confocal fluorescence microscopy imaging, cells were fixed with 10% formalin (HistoFix, Roth) and were then stained with Acti-Stain 670 (Cytoskeleton) and DAPI (RotiMount, Roth) according to the manufacturer’s instructions. Imaging was performed using the Stellaris 8 confocal microscope (Leica) at the Unit for Advanced Light and Electron Microscopy, Center for Biological Threats and Special Pathogens at the Robert Koch Institute. Image processing was performed using ImageJ software.

### 4.5 Real-time quantitative PCR

Viral RNA levels in cell culture supernatants were measured using real-time quantitative PCR. In brief, 140 µL of cell culture supernatant were collected per sample from mock-, MARV- and SUDV-infected bmMΦs at indicated timepoints of infection and were added to 560 µL of AVL buffer (Qiagen). For sample inactivation, 560 µL of 100% ethanol was added to the sample-AVL mix for removal from the BSL4 facility following approved SOPs by trained personnel. RNA from these samples was extracted using the QIAamp Viral RNA Kit (Qiagen) following the manufacturer’s instructions. MARV and SUDV transcripts were quantified using a qPCR assay targeting the NP gene of each virus using an AgPath-ID One-Step RT-PCR Kit (Thermo Fischer Scientific). 25 µL reactions were formulated by adding 5 µL of sample into a master mix containing 10 µM of forward and reverse primers, 10 µM of TaqMan probe, 1x buffer, and 1x RT-PCR enzyme mix. The thermal profile used a 15 min incubation at 45°C, a 10 min incubation at 95°C, and 45 cycles of 15 s at 95°C, followed by 60 s at 60°C. Sample CT values for each virus were compared to a standard curve using MARV or SUDV transcripts of known concentrations ranging from 10^1^ to 10^6^ copies. Viral gene copies per µL cell culture supernatant were then calculated based on the standard curves. The primer and probe sequences used in this study are provided in **Supplementary Table S1**.

### 4.6 Gene expression analysis

For bulk RNA sequencing (RNAseq) of mock-infected, LPS-treated and virus-infected bmMΦs from four biological replicates, adherent cells were lysed in 350 μL/well of RLT buffer. The cell-RLT mixture was then transferred in clean 2 mL sample tubes and were inactivated by adding 600 μL of 70% ethanol to the sample-RLT mix. Following inactivation, samples were removed from the BSL4 facility by trained scientific staff following approved SOPs. Total RNA was extracted using the QIAGEN RNeasy Mini Kit following the manufacturer’s instructions. Extracted RNA samples were submitted to Novogene for library preparation, quality control and sequencing. In brief, messenger RNA (mRNA) was purified from total RNA using poly-T oligo-attached magnetic beads. Following fragmentation, the first strands of complementary DNAs (cDNA) were synthesized using random hexamer primers, followed by the second cDNA strand synthesis, end repair, A-tailing, adapter ligation, size selection, amplification, and purification. After final quality control, cDNA libraries were sequenced on multiple lanes using an Illumina NovaSeq platform.

The final RNAseq reads underwent quality control using FastQC (80). Index adaptors were trimmed using Trim Galore and low-quality base-calls or reads below 20 base pairs were removed using a read quality cutoff Phred score of 33 (81). Trimmed quality-controlled reads were merged into a single file for each sample and were aligned against the *R. aegyptiacus* mRouAeg1.p reference genome (GenBank accession number GCA_014176215.1). For viral gene counts, trimmed and filtered reads were aligned against either the MARV ViralProj15199 (GenBank accession number GCF_000857325.2) or the SUDV ViralProj15012 (GenBank accession number GCF_000855585.1) reference genome. Gene level counts were quantified using Kallisto (82), followed by filtering and log_2_ normalization of gene counts using the tidyverse, baseR and edgeR packages in RStudio (83, 84). Differential gene expression analysis was performed using the Bioconductor package DESEQ2 to identify genes differentially expressed between mock-infected, LPS-treated and virus-infected bmMΦs (85). Differentially expressed genes (DEGs) were defined as having a *p-adj* value < 0.05 and a log_2_-fold change expression of >1.5 for upregulated or <-1.5 for downregulated genes. P-value adjustment (*p-adj*) was automatically calculated by the DESEQ2 algorithm from the Wald test and is corrected for multiple testing using the Benjamini and Hochberg method.

### 4.7 Correlation analysis of gene expression

To assess the possibility of differential host immune response antagonism by viral proteins with known immunosuppressive properties, the log_2_-fold change values of MARV-VP35, MARV-VP40, SUDV-VP24 and SUDV-VP35 were extracted in a separate table. The complete list of DEGs was then manually inspected to identify and extract into the same table the log_2_-fold change values of all type I and type III IFN response-associated genes differentially expressed in virus-infected ERB at days 1, 2 and 3 post-infection. The complete table containing all viral protein and IFN gene expressions across the three timepoints was loaded in RStudio. Spearman’s rank correlation coefficient analysis was then performed for every gene against every other gene separately for MARV-infected and SUDV-infected samples using the corrr and corrplot RStudio packages. Significance levels were set at *p* < 0.05 and insignificant correlations were blanked out from the pyramid tables using corrplot.

### 4.8 Ingenuity pathway analysis

Significantly enriched pathways and upstream regulators were determined using Ingenuity Pathway Analysis (IPA, QIAGEN Digital Insights, Redwood City, CA, USA) for each timepoint for SUDV and MARV. Datasets for both ERB and human cells included DEG log_2_-fold change values and *p-adj* values at each time point and analyzed using “Core Analysis” with default settings. Log_2_-fold change and *p-adj* values were calculated and corrected by DESEQ2 as described above. Subsequent “Comparison Analysis” with default settings was performed to find commonalties in pathway enrichment and upstream regulators across timepoints for both viruses. Canonical pathways are ranked using a z-score algorithm that is calculated based upon dataset correlation from the uploaded DEG and *p-adj* values with an activated state in that canonical pathway. P-values result from a Fischer’s exact test that calculates the probability that the association between the genes in the uploaded dataset and the genes in the canonical pathway are due to chance alone. The pathway map in **Figure 7** was modified from the “Pathogen Induced Cytokine Storm Signaling Pathway” figure by removing downstream connections not relevant to the study. The heatmaps of canonical pathway and upstream regulator expression were created using GraphPad Prism 9 software (CA, USA).

### 4.9 CIBERSORTx analysis

To calculate the cell subset composition of the ERB bmMΦ cultures, normalized log2 gene counts of mock-infected cells from individual bats were uploaded for analysis in the web-based tool Cell-type Identification by Estimating Relative Subsets of RNA Transcripts (CIBERSORTx, https://cibersort.stanford.edu/, Stanford University, Stanford, CA, USA). The analysis was performed using a human reference set of 22 immune cell subtypes as a signature matrix and was run for 100 permutations. The results were visualized using Graphpad Prism version 9.1.0 (GraphPad Prism Software, Inc., La Jolla, CA, USA).

### 4.10 Statistical analysis

Flow cytometric data were tested for normality using the Shapiro-Wilk normality test, followed by a Wilcoxon Signed Rank test (**Figure 1D**) or a Šídák’s multiple comparison test (**Figure 1E**). Viral gene copy results were tested for significance using Tukey’s multiple comparison’s test (**Figures 2B–E**). Log_2_-fold change values of host DEGs following bulk RNA sequencing were based on Wald tests for differential expression and were defined as having a *p-adj* value < 0.05 and a log_2_-fold change expression of >1.5 for upregulated or <-1.5 for downregulated genes (85). All *p-adj* values associated with differential gene expression were automatically calculated by the DESEQ2 algorithm from the Wald test and were corrected for multiple testing using the Benjamini and Hochberg method. Gene expression correlation analyses were performed using the non-parametric Spearman’s rank correlation coefficient test, with a significance cut-off value set at *p* < 0.05. The results for virus progeny in cell culture supernatants comparing ERB and human MΦs were tested for normality using the Shapiro-Wilk normality test, followed by the Mann-Whitney-U-test (**Figure 8B**). The results for virus replication in RoNi and Vero E6 cells were tested for normality using the Shapiro-Wilk normality test and their significance levels were tested using an unpaired student’s t-test.

## Data Availability

The RNA sequencing data and metadata presented in this study are available on NCBI under BioProject ID PRJNA1344459 (https://www.ncbi.nlm.nih.gov/bioproject/1344459). The raw sequencing read files are available under accession numbers SAMN52643509, SAMN52643510, SAMN52643511, SAMN52643512, SAMN52643513, SAMN52643514, SAMN52643515, SAMN52643516, SAMN52643517 and SAMN52643518. The code scripts used for the gene expression analysis are available on GitHub (https://github.com/ivetyorda/ERB_bmMac_MARVvsSUDV). All other data presented in this study are available from the corresponding author on reasonable request.

## References

[1] Banerjee A Kulcsar K Misra V Frieman M Mossman K. Bats and coronaviruses. Viruses. (2019) 11:7–9. doi: 10.3390/v11010041, PMID: 30634396 PMC6356540

[2] Crowley D Becker D Washburne A Plowright R. Identifying suspect bat reservoirs of emerging infections. Vaccines (Basel). (2020) 8:1–11. doi: 10.3390/vaccines8020228, PMID: 32429501 PMC7349958

[3] Soler-Tovar D Escobar LE. Rabies transmitted from vampires to cattle: An overview. PLoS One. (2025) 20:1–18. doi: 10.1371/journal.pone.0317214, PMID: 39804921 PMC11730393

[4] Banerjee A Misra V Schountz T Baker ML. Tools to study pathogen-host interactions in bats. Virus Res. (2018) 248:5–12. doi: 10.1016/j.virusres.2018.02.013, PMID: 29454637 PMC7114677

[5] Banerjee A Mossman KL Miller MS. Bat influenza viruses: Making a double agent of MHC Class II. Trends microbiol. (2020) 28:703–6. doi: 10.1016/j.tim.2020.04.006, PMID: 32409145 PMC7167561

[6] Irving AT Ahn M Goh G Anderson DE Wang LF. Lessons from the host defenses of bats, a unique viral reservoir. Nature. (2021) 589:363–70. doi: 10.1038/s41586-020-03128-0, PMID: 33473223

[7] Baid K Irving AT Jouvenet N Banerjee A. The translational potential of studying bat immunity. Trends Immunol. (2024) 45:188–97. doi: 10.1016/j.it.2024.01.007, PMID: 38453577

[8] Gonzalez V Hurtado-Monzón AM O’Krafka S Mühlberger E Letko M Frank HK. Studying bats using a One Health lens: Bridging the gap between bat virology and disease ecology. J Virol. (2024) 98:e01453024. doi: 10.1128/jvi.01453-24, PMID: 39499009 PMC11650978

[9] Morales AE Dong Y Brown T Baid K Kontopoulos DG Gonzalez V. Bat genomes illuminate adaptations to viral tolerance and disease resistance. Nature. (2025) 638:449–58. doi: 10.1038/s41586-024-08471-0, PMID: 39880942 PMC11821529

[10] Yan X Liu Y Hu T Huang Z Li C Guo L. A compendium of 8,176 bat RNA viral metagenomes reveals ecological drivers and circulation dynamics. Nat Microbiol. (2025) 10:554–68. doi: 10.1038/s41564-024-01884-7, PMID: 39833544

[11] Towner JS Amman BR Sealy TK Reeder Carrol SA Comer JA Kemp A. Isolation of genetically diverse Marburg viruses from Egyptian fruit bats. PLoS Pathog. (2009) 5:e1000536. doi: 10.1371/journal.ppat.1000536, PMID: 19649327 PMC2713404

[12] Schuh AJ Amman BR Sealy TK Spengler JR Nichol ST. Egyptian rousette bats maintain long-term protective immunity against Marburg virus infection despite diminished antibody levels. Sci Rep. (2017) 7:8763. doi: 10.1038/s41598-017-07824-2, PMID: 28821722 PMC5562751

[13] Amman BR Schuh AJ Sealy TK Spengler JR Welch SR Kirejczyk SGM. Experimental infection of Egyptian rousette bats (*Rousettus aEgyptiacus*) with Sosuga virus demonstrates potential transmission routes for a bat-borne human pathogenic paramyxovirus. PLoS Negl Trop Dis. (2020) 14:e0008092. doi: 10.1371/journal.pntd.0008092, PMID: 32119657 PMC7067492

[14] Amman BR Carroll SA Reed ZD Sealy TK Balinandi S Swanepoel R. Seasonal pulses of Marburg virus circulation in juvenile *Rousettus aEgyptiacus* bats coincide with periods of increased risk of human infection. PLoS Pathog. (2012) 8:e10002877. doi: 10.1371/journal.ppat.1002877, PMID: 23055920 PMC3464226

[15] Amman BR Jones MEB Sealy TK Uebelhoer LS Schuh AJ Bird BH. Oral shedding of Marburg virus in experimentally infected Egyptian fruit bats (*Rousettus aEgyptiacus*). J Wildl Dis. (2015) 51:113–24. doi: 10.7589/2014-08-198, PMID: 25375951 PMC5022530

[16] Jones MEB Amman BR Sealy TK Uebelhoer LS Schuh AJ Flietstra T. Clinical, histopathologic, and immunohistochemical characterization of experimental Marburg virus infection in a natural reservoir host, the Egyptian rousette bat (*Rousettus aEgyptiacus*). Viruses. (2019) 11:214. doi: 10.3390/v11030214, PMID: 30832364 PMC6466277

[17] Goldstein T Anthony SJ Gbakima A Bird BH Bangura J Tremeau-Bravard A. The discovery of Bombali virus adds further support for bats as hosts of ebolaviruses. Nat Microbiol. (2018) 3:1084–9. doi: 10.1038/s41564-018-0227-2, PMID: 30150734 PMC6557442

[18] Edenborough KM Bokelmann M Lander A Couacy-Hymann E Lechner J Drechsel O. Dendritic cells generated from *Mops condylurus*, a likely filovirus reservoir host, are susceptible to and activated by Zaire ebolavirus infection. Front Immunol. (2019) 10:2414. doi: 10.3389/fimmu.2019.02414, PMID: 31681302 PMC6797855

[19] Riesle-Sbarbaro SA Wibbelt G Düx A Kouakou V Bokelmann M Hansen-Kant K. Selective replication and vertical transmission of Ebola virus in experimentally infected Angolan free-tailed bats. Nat Commun. (2024) 15:925. doi: 10.1038/s41467-024-45231-0, PMID: 38297087 PMC10830451

[20] Marí Saéz A Weiss S Nowak K Lapeyre V Zimmermann F Düx A. Investigating the zoonotic origin of the West African Ebola epidemic. EMBO Mol Med. (2015) 7:17–23. doi: 10.15252/emmm.201404792, PMID: 25550396 PMC4309665

[21] Brauburger K Hume AJ Mühlberger E Olejnik J. Forty-five years of Marburg virus research. Viruses. (2012) 4:1878–927. doi: 10.3390/v4101878, PMID: 23202446 PMC3497034

[22] Marzi A Feldmann H. Marburg Virus Disease: Global threat or isolated events? J Infect Dis. (2023) 228:103–5. doi: 10.1093/infdis/jiad161, PMID: 37183957 PMC10345466

[23] Malvy D McElroy AK de Clerck H Günther S van Griensven J. Ebola virus disease. Lancet. (2019) 393:936–48. doi: 10.1016/S0140-6736(18)33132-5, PMID: 30777297

[24] Fontana L Ondo Avomo CO Ngomo Mikue LE Fuga Eyemam DN Nguere MA Mometolo IA. Case series of patients with Marburg Virus Disease, Equatorial Guinea, 2023. NEJM. (2024) 391:283–5. doi: 10.1056/NEJMc2313181, PMID: 39018539 PMC11976099

[25] Baseler L Chertow DS Johnson KM Feldmann H Morens DM. The pathogenesis of ebola virus disease. Ann Rev Pathol. (2017) 12:387–418. doi: 10.1146/annurev-pathol-052016-100506, PMID: 27959626

[26] Jones MEB Schuh AJ Amman BR Sealy TK Zaki SR Nichol ST. Experimental inoculation of Egyptian rousette bats (*Rousettus aEgyptiacus*) with viruses of the ebolavirus and marburgvirus genera. Viruses. (2015) 7:3420–42. doi: 10.3390/v7072779, PMID: 26120867 PMC4517108

[27] Guito JC Kirejczyk SGM Schuh AJ Amman BR Sealy TK Graziano J. Coordinated inflammatory responses dictate Marburg virus control by reservoir bats. Nat Commun. (2024) 15:1826. doi: 10.1038/s41467-024-46226-7, PMID: 38418477 PMC10902335

[28] Prescott J Guito JC Spengler JR Arnold CE Schuh AJ Amman BR. Rousette bat dendritic cells overcome Marburg virus-mediated antiviral responses by upregulation of interferon-related genes while downregulating proinflammatory disease mediators. mSphere. (2019) 4:1–14. doi: 10.1128/mSphere.00728-19, PMID: 31801842 PMC6893212

[29] Guito JC Prescott JB Arnold CE Palacios GF Towner JS. Asymptomatic infection of Marburg virus reservoir bats is explained by a strategy of immunoprotective disease tolerance. Curr Biol. (2021) 31:257–270.e5. doi: 10.1016/j.cub.2020.10.015, PMID: 33157026

[30] Bosio CM Aman MJ Grogan C Hogan R Ruthel G Negley D. Ebola and Marburg viruses replicate in monocyte-derived dendritic cells without inducing the production of cytokines and full maturation. J Infect Dis. (2003) 188:1630–8. doi: 10.1086/379199, PMID: 14639532

[31] Olejnik J Ryabchikova E Corley RB Mühlberger E. Intracellular events and cell fate in filovirus infection. Viruses. (2011) 3:1501–31. doi: 10.3390/v3081501, PMID: 21927676 PMC3172725

[32] Yen B Mulder LCF Martinez O Basler CF. Molecular basis for Ebolavirus VP35 suppression of human dendritic cell maturation. J Virol. (2014) 88:12500–10. doi: 10.1128/JVI.02163-14, PMID: 25142601 PMC4248944

[33] Yen BC Basler CF. Effects of filovirus interferon antagonists on responses of human monocyte-derived dendritic cells to RNA virus infection. J Virol. (2016) 90:5108–18. doi: 10.1128/JVI.00191-16, PMID: 26962215 PMC4859717

[34] Olejnik J Forero A Deflubé LR Hume AJ Manhart WA Nishida A. Ebolaviruses associated with differential pathogenicity induce distinct host responses in human macrophages. J Virol. (2017) 91:1–22. doi: 10.1128/JVI.00179-17, PMID: 28331091 PMC5432886

[35] Kotliar D Lin AE Logue J Hughes TK Khoury NM Raju SS. Single-cell profiling of ebola virus disease. Vivo reveals Viral Host dynamics. Cell. (2020) 183:1383–1401.e19. doi: 10.1016/j.cell.2020.10.002, PMID: 33159858 PMC7707107

[36] Yordanova IA Lander A Wahlbrink A Towner JS Albariño CG Ang LT. Human macrophages infected with Egyptian rousette bat-isolated Marburg virus display inter-individual susceptibility and antiviral responsiveness. NPJ Viruses. (2024) 2:19. doi: 10.1038/s44298-024-00027-3, PMID: 40295691 PMC11721647

[37] Albariño CG Guerrero LW Chakrabarti AK Nichol ST. Transcriptional analysis of viral mRNAs reveals common transcription patterns in cells infected by five different filoviruses. PLoS One. (2018) 13:e0201827. doi: 10.1371/journal.pone.0201827, PMID: 30071116 PMC6072132

[38] Dolnik O Becker S. Assembly and transport of filovirus nucleocapsids. PLoS Pathog. (2022) 18:e1010616. doi: 10.1371/journal.ppat.1010616, PMID: 35900983 PMC9333320

[39] Kainulainen MH Harmon JR Whitesell AN Bergeron É Karaaslan E Cossaboom CM. Recombinant Sudan virus and evaluation of humoral cross-reactivity between Ebola and Sudan virus glycoproteins after infection or rVSV-ΔG-ZEBOV-GP vaccination. Emerg Microb Infect. (2023) 12:2265660. doi: 10.1080/22221751.2023.2265660, PMID: 37787119 PMC10623891

[40] Krähling V Dolnik O Kolesnikova L Schmidt-Chanasit J Jordan I Sandig V. Establishment of fruit bat cells (*Rousettus aEgyptiacus*) as a model system for the investigation of filoviral infection. PLoS Negl Trop Dis. (2010) 4:e802. doi: 10.1371/journal.pntd.0000802, PMID: 20808767 PMC2927428

[41] Pavlovich SS Lovett SP Koroleva G Guito JC Arnold CE Nagle ER. The Egyptian rousette genome reveals unexpected features of bat antiviral immunity. Cell. (2018) 173:1098–1110.e18. doi: 10.1016/j.cell.2018.03.070, PMID: 29706541 PMC7112298

[42] Moreno Santillán DD Lama TM Gutierrez Guerrero YT Brown AM Donat P Zhao H. Large-scale genome sampling reveals unique immunity and metabolic adaptations in bats. Mol Ecol. (2021) 30:6449–67. doi: 10.1111/mec.16027, PMID: 34146369

[43] Cruz-Rivera PCD Eitson JL Schoggins JW. IRF7 from the black flying fox induces a STAT1-independent ISG response in unstimulated cell lines that protects against diverse RNA viruses. bioRxiv. (2024). doi: 10.1101/2024.05.02.592239, PMID: 38746207 PMC11092574

[44] Zhou P Chionh YT Irac SE Ahn M Ng JHJ Fossum E. Unlocking bat immunology: Establishment of *Pteropus alecto* bone marrow-derived dendritic cells and macrophages. Sci Rep. (2016) 6:1–10. doi: 10.1038/srep38597, PMID: 27934903 PMC5146944

[45] Gamage AM Zhu F Ahn M Foo RJH Hey YY Low DHW. Immunophenotyping monocytes, macrophages and granulocytes in the Pteropodid bat *Eonycteris* sp*elaea* . Sci Rep. (2020) 10:1–16. doi: 10.1038/s41598-019-57212-1, PMID: 31941952 PMC6962400

[46] Ruthel G Demmin GL Kallstrom G Javid MP Badie SS Will AB. Association of Ebola virus matrix protein VP40 with microtubules. J Virol. (2005) 79:4709–19. doi: 10.1128/JVI.79.8.4709-4719.2005, PMID: 15795257 PMC1069569

[47] Acharya D Reis R Volcic M Liu GQ Wang MK Chia BS. Actin cytoskeleton remodeling primes RIG-I-like receptor activation. Cell. (2022) 185:3588–3602.e21. doi: 10.1016/j.cell.2022.08.011, PMID: 36113429 PMC9680832

[48] Noda T Ebihara H Muramoto Y Fujii K Takada A Sagara H. Assembly and budding of ebolavirus. PLoS Pathog. (2006) 2:0864–72. doi: 10.1371/journal.ppat.0020099, PMID: 17009868 PMC1579243

[49] Ploubidou A Way M. Viral transport and the cytoskeleton. Curr Op Cell Biol. (2001) 13:97–105. doi: 10.1016/s0955-0674(00)00180-0, PMID: 11163140 PMC7125730

[50] Serazev TV Nadezhdina ES Shanina NA Leshchiner AD Kalinina NO Morozov SY. Virions and the coat protein of the potato virus X interact with microtubules and induce tubulin polymerization. vitro. Mol Biol. (2003) 37:919–25. doi: 10.1023/B:MBIL.0000008362.88344.f3 14714504

[51] Kolesnikova L Bohil AB Cheney RE Becker S. Budding of Marburgvirus is associated with filopodia. Cell Microbiol. (2007) 9:939–51. doi: 10.1111/j.1462-5822.2006.00842.x, PMID: 17140405

[52] Valmas C Grosch MN Schümann M Olejnik J Martinez O Best SM. Marburg virus evades interferon responses by a mechanism distinct from Ebola virus. PLoS Pathog. (2010) 6:e1000721. doi: 10.1371/journal.ppat.1000721, PMID: 20084112 PMC2799553

[53] Ramanan P Edwards MR Shabman RS Leung DW Endlich-Frazier AC. Structural basis for Marburg virus VP35–mediated immune evasion mechanisms. Proc Nat Acad Sci. (2012) 109:20661–6. doi: 10.1073/pnas.1213559109, PMID: 23185024 PMC3528546

[54] Albariño CG Guerrero LW Spengler JR Uebelhoer LS Chakrabarti AK Nichol ST. Recombinant Marburg viruses containing mutations in the IID region of VP35 prevent inhibition of host immune responses. Virol. (2015) 476:85–91. doi: 10.1016/j.virol.2014.12.002, PMID: 25531184 PMC6461211

[55] Edwards MR Liu G Mire CE Sureshchandra S Luthra P Yen B. Differential regulation of interferon responses by Ebola and Marburg virus VP35 proteins. Cell Rep. (2016) 14:1632–40. doi: 10.1016/j.celrep.2016.01.049, PMID: 26876165 PMC4767585

[56] Guito JC Albariño CG Chakrabarti AK Towner JS. Novel activities by ebolavirus and marburgvirus interferon antagonists revealed using a standardized. Vitro Rep system. Virol. (2017) 501:147–65. doi: 10.1016/j.virol.2016.11.015, PMID: 27930961 PMC11524407

[57] Kimberlina CR Bornholdt ZA Li S Woods VL MacRae IJ Saphire EO. Ebolavirus VP35 uses a bimodal strategy to bind dsRNA for innate immune suppression. Proc Nat Acad Sci. (2010) 107:314–9. doi: 10.1073/pnas.0910547107, PMID: 20018665 PMC2806767

[58] Leung DW Prins KC Borek DM Farahbakhsh M Tufariello JM Ramanan P. Structural basis for dsRNA recognition and interferon antagonism by Ebola VP35. Nat Struct Mol Biol. (2010) 17:165–72. doi: 10.1038/nsmb.1765, PMID: 20081868 PMC2872155

[59] Prins KC Binning JM Shabman RS Leung DW Amarasing GK Basler CF. Basic Residues within the Ebolavirus VP35 protein are required for its viral polymerase cofactor function. J Virol. (2010) 84:10581–91. doi: 10.1128/JVI.00925-10, PMID: 20686031 PMC2950600

[60] Hartman AL Bird BH Towner JS Antoniadou ZA Zaki SR Nichol ST. Inhibition of IRF-3 Activation by VP35 is critical for the high level of virulence of Ebola virus. J Virol. (2008) 82:2699–704. doi: 10.1128/JVI.02344-07, PMID: 18199658 PMC2259001

[61] Arnold CE Guito JC Altamura LA Lovett SP Nagle ER Palacios GF. Transcriptomics reveal antiviral gene induction in the Egyptian rousette bat is antagonized *in vitro* by Marburg virus infection. Viruses. (2018) 10:607. doi: 10.3390/v10110607, PMID: 30400182 PMC6266330

[62] Mandl JN Schneider C Schneider DS Baker ML. Going to bat(s) for studies of disease tolerance. Front Immunol. (2018) 9:2112. doi: 10.3389/fimmu.2018.02112, PMID: 30294323 PMC6158362

[63] Solstad AD Denz PJ Kenney AD Mahfooz NS Speaks S Gong Q. IFN-λ uniquely promotes CD8 T cell immunity against SARS-CoV-2 relative to type I IFN. JCI Insight. (2024) 9:1–16. doi: 10.1172/jci.insight.171830, PMID: 38973611 PMC11383353

[64] Teijaro JR. Type I interferons in viral control and immune regulation. Curr Op Virol. (2016) 16:31–40. doi: 10.1016/j.coviro.2016.01.001, PMID: 26812607 PMC4821698

[65] Sadler AJ Williams BRG. Interferon-inducible antiviral effectors. Nat Rev Immunol. (2008) 8:559–68. doi: 10.1038/nri2314, PMID: 18575461 PMC2522268

[66] Hoffmann HH Schneider WM Rice CM. Interferons and viruses: An evolutionary arms race of molecular interactions. Trends Immunol. (2015) 36:124–38. doi: 10.1016/j.it.2015.01.004, PMID: 25704559 PMC4384471

[67] Syedbasha M Egli A. Interferon Lambda: Modulating immunity in infectious diseases. Front Immunol. (2017) 8:119. doi: 10.3389/fimmu.2017.00119, PMID: 28293236 PMC5328987

[68] Zhou P Tachedjian M Wynne JW Boyd V Cui J Smith I. Contraction of the type I IFN locus and unusual constitutive expression of IFN-α in bats. Proc Nat Acad Sci. (2016) 113:2696–701. doi: 10.1073/pnas.1518240113, PMID: 26903655 PMC4790985

[69] Kepler TB Sample C Hudak K Roach J Haines A. Chiropteran types I and II interferon genes inferred from genome sequencing traces by a statistical gene-family assembler. BMC Genom. (2010) 11:444. doi: 10.1186/1471-2164-11-444, PMID: 20663124 PMC3091641

[70] Geng R Wang Q Yao YL Shen XR Jia JK Wang X. Unconventional IFNω -like genes dominate the type I IFN locus and the constitutive antiviral responses in bats. J Immunol. (2024) 213:204–13. doi: 10.4049/jimmunol.2300301, PMID: 38856712

[71] Kellner MJ Monteil VM Zelger P Pei G Jiao J Onji M. Bat organoids reveal antiviral responses at epithelial surfaces. Nat Immunol. (2025) 26:934–46. doi: 10.1038/s41590-025-02155-1, PMID: 40399606 PMC12133567

[72] Hutchinson KL Rollin PE. Cytokine and chemokine expression in humans infected with Sudan Ebola virus. J Infect Dis. (2007) 196:357–63. doi: 10.1086/520611, PMID: 17940971

[73] Gupta M MacNeil A Reed ZD Rollin PE Spiropoulou CF. Serology and cytokine profiles in patients infected with the newly discovered Bundibugyo ebolavirus. Virol. (2012) 423:119–24. doi: 10.1016/j.virol.2011.11.027, PMID: 22197674

[74] Greenberg A Huber BR Liu DX Logue JP Hischak AMW Hart RJ. Quantification of viral and host biomarkers in the liver of rhesus macaques: a longitudinal study of Zaire Ebolavirus strain Kikwit (EBOV/Kik). Am J Pathol. (2020) 190:1449–60. doi: 10.1016/j.ajpath.2020.03.003, PMID: 32275904 PMC7322367

[75] Vernet MA Reynard S Fizet A Schaeffer J Pannetier D Guedj J. Clinical, virological, and biological parameters associated with outcomes of Ebola virus infection in Macenta, Guinea. JCI Insight. (2017) 2:1–14. doi: 10.1172/jci.insight.88864, PMID: 28352651 PMC5358491

[76] Fernando L Qiu X Melito PL Williams KJN Feldmann F Feldmann H. Immune response to Marburg virus Angola infection in nonhuman primates. J Infect Dis. (2015) 212:S234–41. doi: 10.1093/infdis/jiv095, PMID: 25957966

[77] Yaddanapudi K Palacios G Towner JS Chen I Sariol CA Nichol ST. Implication of a retrovirus-like glycoprotein peptide in the immunopathogenesis of Ebola and Marburg viruses. FASEB J. (2006) 20:2519–30. doi: 10.1096/fj.06-6151com, PMID: 17023517

[78] Wiedemann A Foucat E Hocini H Lefebvre C Hejblum BP Durand M. Long-lasting severe immune dysfunction in Ebola virus disease survivors. Nat Commun. (2020) 11:1–11. doi: 10.1038/s41467-020-17489-7, PMID: 32709840 PMC7381622

[79] Guito JC Arnold CE Schuh AJ Amman BR Sealy TK Spengler JR. Peripheral immune responses to filoviruses in a reservoir versus spillover hosts reveal transcriptional correlates of disease. Front Immunol. (2024) 14:1306501. doi: 10.3389/fimmu.2023.1306501, PMID: 38259437 PMC10800976

[80] Andrews S. FastQC: A Quality Control Tool for High Throughput Sequence Data (2010). Available online at: http://www.bioinformatics.babraham.ac.uk/projects/fastqc/ (Accessed August 15, 2025).

[81] Krueger F. Trim Galore (2012). Available online at: https://www.bioinformatics.babraham.ac.uk/projects/trim_galore/ (Accessed August 15, 2025).

[82] Bray NL Pimentel H Melsted P Pachter L. Near-optimal probabilistic RNA-seq quantification. Nat Biotechnol. (2016) 34:525–7. doi: 10.1038/nbt.3519, PMID: 27043002

[83] Robinson MD McCarthy DJ Smyth GK. edgeR: A Bioconductor package for differential expression analysis of digital gene expression data. J Bioinform. (2009) 26:139–40. doi: 10.1093/bioinformatics/btp616, PMID: 19910308 PMC2796818

[84] Wickham H Averick M Bryan J Chang W McGowan LDA François R. Welcome to the tidyverse. J Open Source Software. (2019) 4:1686. doi: 10.21105/joss.01686

[85] Love MI Huber W Anders S. Moderated estimation of fold change and dispersion for RNA-seq data with DESeq2. Genome Biol. (2014) 15:550. doi: 10.1186/s13059-014-0550-8, PMID: 25516281 PMC4302049

