# Supplementary material for: Marburg and Sudan viruses elicit divergent interferon responses and cytokine storm signaling in Egyptian rousette bat macrophages
Source: Front Immunol. 2025 Oct 17;16:1686343. doi: 10.3389/fimmu.2025.1686343 (PMC12575105; doi:10.3389/fimmu.2025.1686343)
Supplement: Supplementary file 1 [file DataSheet1.docx]

Supplementary Material


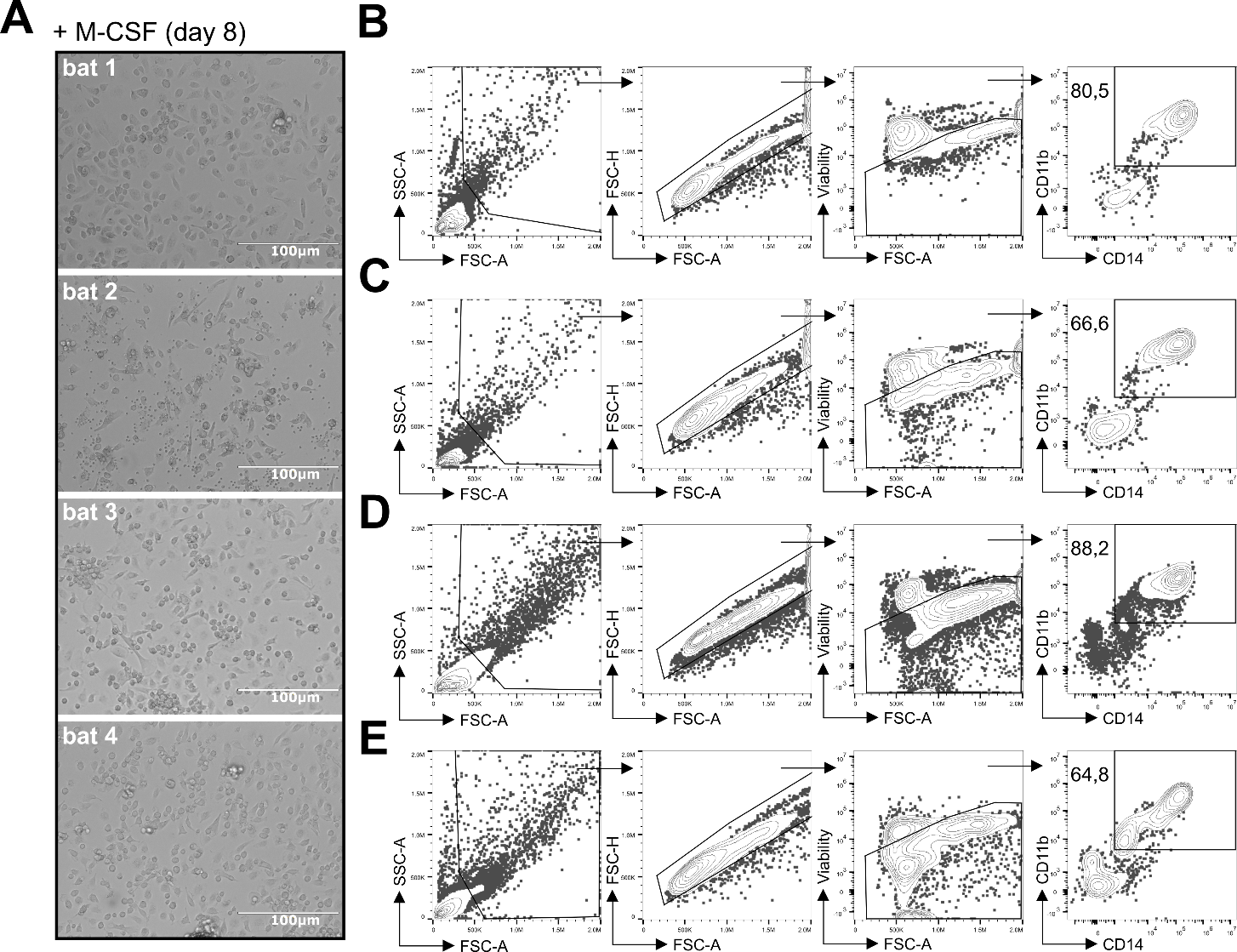


**Supplementary Figure 1.** Differentiation of Egyptian rousette bat (ERB) bone marrow-derived macrophages (bmMΦs). **(A)** Example light microscopy images illustrating the morphology of bone marrow cell cultures after 8 days of *in vitro* differentiation with recombinant ERB-specific M-CSF using cells derived from four individual bats. **(B-E)** Corresponding gating strategies and example contour plots showing the identification of CD11b^+^CD14^+^ bmMΦs via flow cytometry from four individual bats.


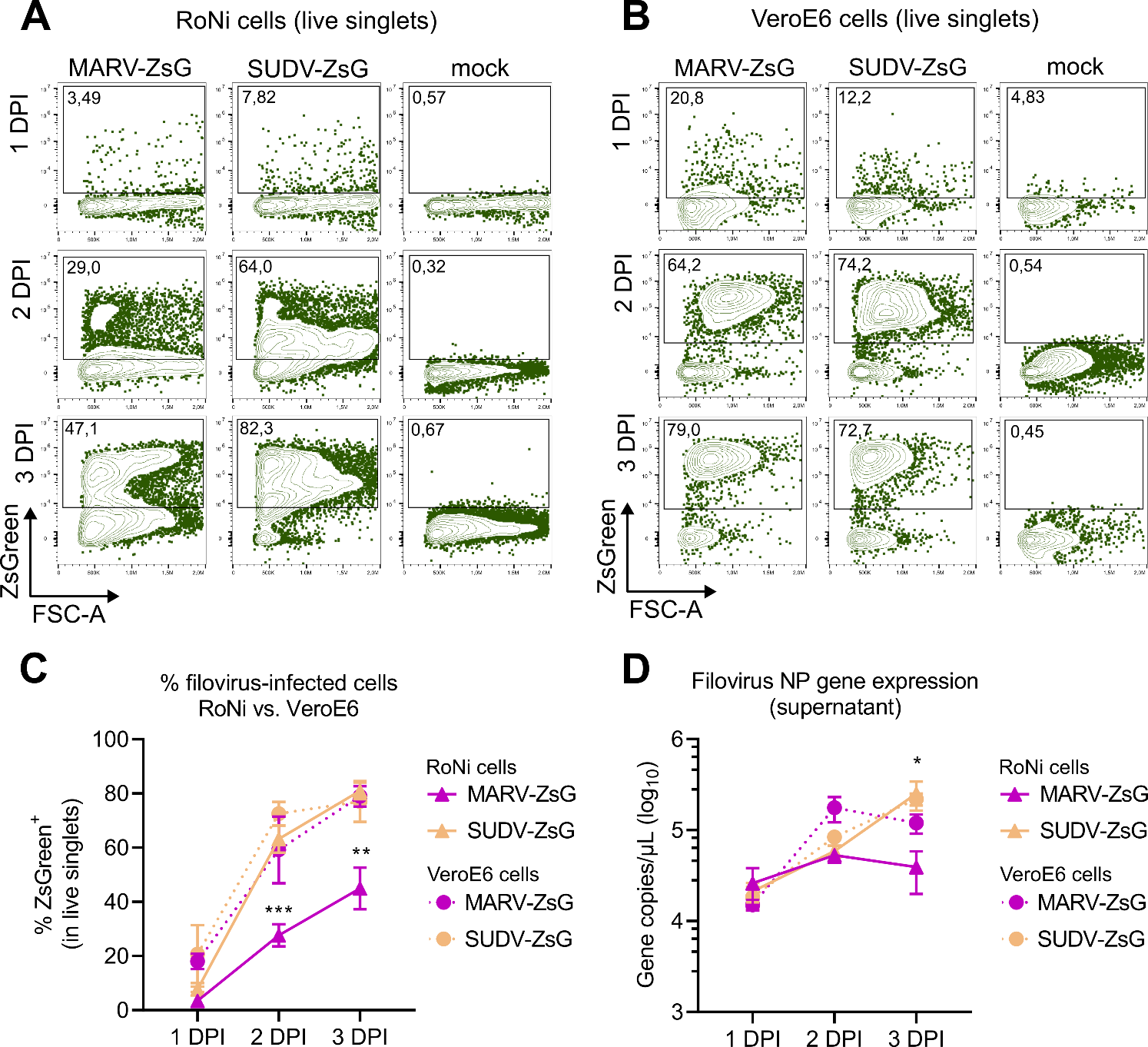


**Supplementary Figure 2.** Filovirus replication in RoNi and Vero E6 cell lines. Example contour plots of ZsGreen signal in live singlets in **(A)** RoNi cells and **(B)** Vero E6 cells infected with either MARV-ZsG or SUDV-ZsG, measured on a flow cytometer at 1, 2 and 3 DPI. **(C)** The percentage ZsGreen^+^ cells in live singlets and **(D)** Filovirus NP gene copies in cell culture supernatants of MARV-ZsG and SUDV-ZsG infected RoNi and Vero E6 cells at 1, 2 and 3 DPI. RoNi and Vero E6 infection experiments were performed using three technical replicates for each cell type, virus and timepoint of infection. Statistical analysis in **(C)** and **(D)** was performed using an unpaired student’s t-test. Statistical significance stars mark significant differences between MARV-ZsG and SUDV-ZsG in RoNi cells. The graphs in (**C**) and (**D**) illustrate mean ± SD for each group. **p*<0.05, ***p*<0.01, ****p*<0.001.


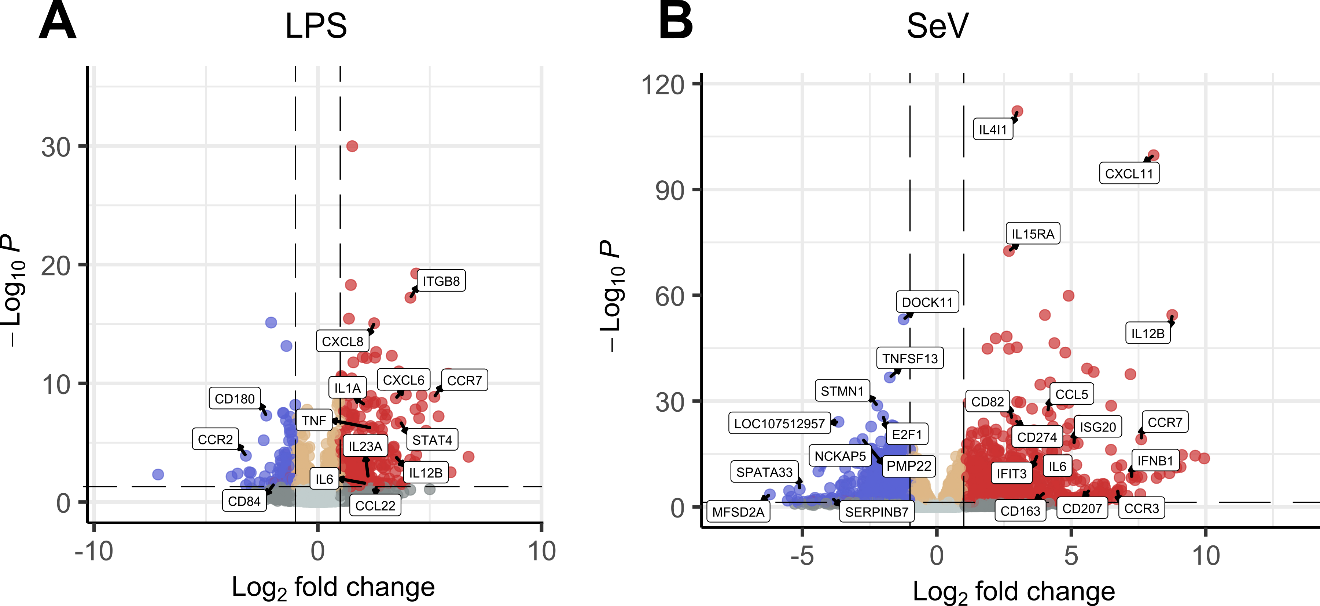


**Supplementary Figure 3.** ERB-derived bmMΦ responses to general immune stimulation. Volcano plots of significant DEGs across cells from four individual bats in **(A)** LPS-stimulated and **(B)** SeV-infected bmMΦs, compared with mock-infected controls. DEGs were defined as genes with a *p-adj* < 0.05 and a log_2_-fold change ≥ ± 1.5. Genes with log_2_-fold change ≥ 1.5 are marked in red (upregulated DEGs), genes with log_2_-fold change ≥ -1.5 are marked in blue (downregulated DEGs). Genes with a log_2_-fold change < ± 1.5 are marked in yellow, while genes with a *p-adj* > 0.05 are marked in grey (non-DEGs).


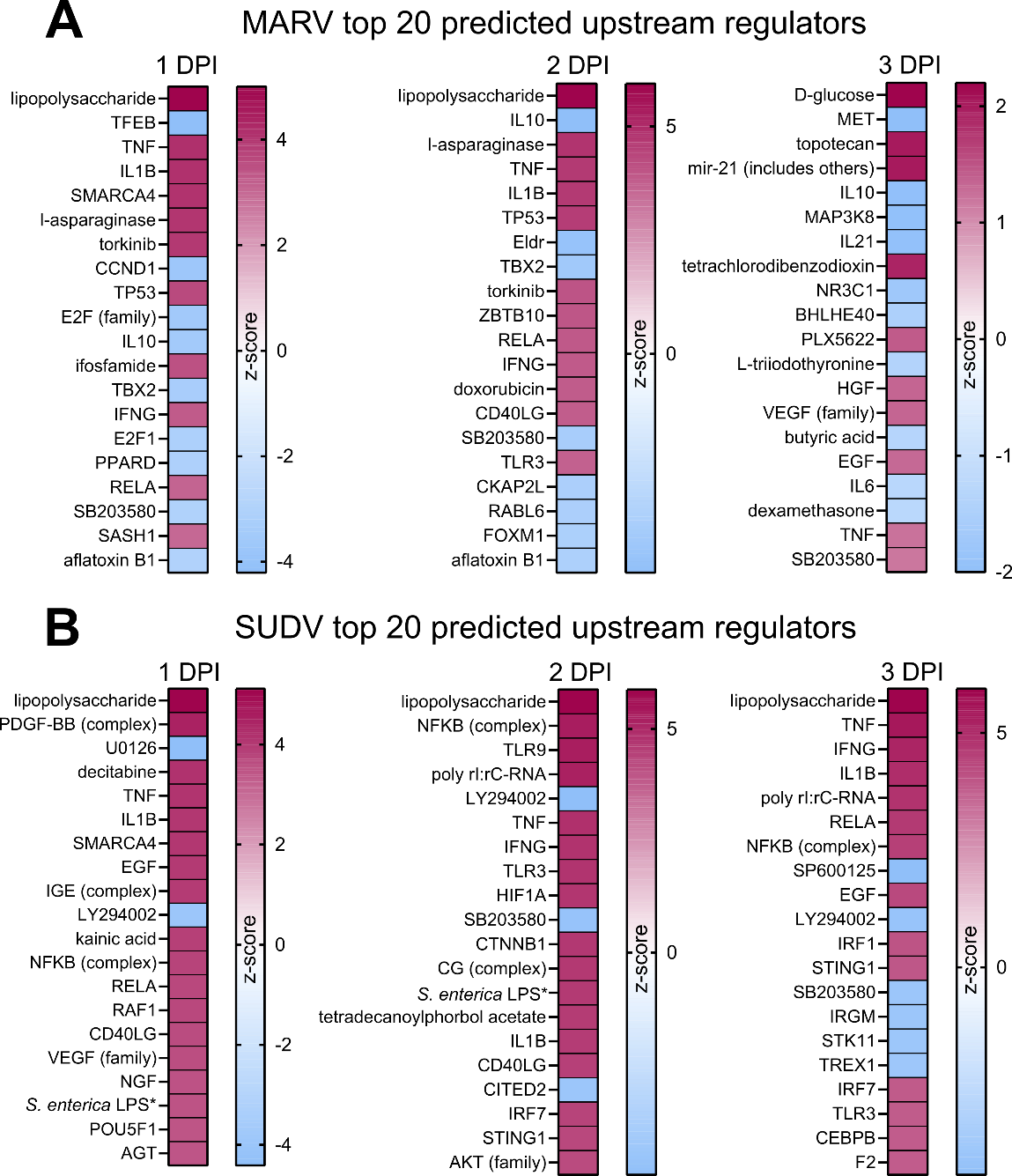


**Supplementary Figure 4.** Differential regulation of signaling pathways in filovirus-infected ERB bmMΦs. Ingenuity Pathway Analysis (IPA) of the statistically significant (*p*<0.05) **(A)** Top 20 predicted upstream regulators upregulated (magenta) or downregulated (blue) in response to MARV at 1, 2 and 3 DPI, and **(B)** Top 20 predicted upstream regulators upregulated (magenta) or downregulated (blue) in response to SUDV at 1, 2 and 3 DPI.

**Supplementary Table 1.** Real-time quantitative PCR primer and probe sequences for quantification of viral RNA copies in cell culture supernatants, targeting MARV-NP and SUDV-NP.

| **Virus** | **Forward primer** | **Reverse primer** | **TaqMan probe** |
| --- | --- | --- | --- |
| MARV | GTCCTCAGCCAGAAACGAGA | ACCGTTACTTCCACAGGTGT | 6Fam-TCACAGAATCGGGTGTCACAGTCGT-BBQ |
| SUDV | GGTGGTGTTGTTGACCCGTA | CATCGTCGTCGTCCAAATTGA | 6Fam-TGAAGGCACCACAGGAGATCTTGATCT-BBQ |
